# iNOS aggravates pressure overload-induced cardiac dysfunction via activation of the cytosolic-mtDNA-mediated cGAS-STING pathway

**DOI:** 10.7150/thno.84049

**Published:** 2023-07-24

**Authors:** Yongzheng Guo, Yuehua You, Fei-Fei Shang, Xiaowen Wang, Bi Huang, Boying Zhao, Dingyi Lv, Shenglan Yang, Ming Xie, Lingwen Kong, Dingyuan Du, Suxin Luo, Xin Tian, Yong Xia

**Affiliations:** 1 Division of Cardiology, The First Affiliated Hospital of Chongqing Medical University, Chongqing 400016, China.; 2 Institute of Life Science, Chongqing Medical University, Chongqing 400016, China.; 3 Department of Cardiothoracic Surgery, The First Affiliated Hospital of Chongqing Medical University, Chongqing 400016, China.; 4 Department of Cardiothoracic Surgery, Chongqing University Central Hospital, Chongqing 400014, China.; 5 Department of Neurology, The First Affiliated Hospital of Chongqing Medical University, Chongqing Key Laboratory of Neurology, Chongqing 400016, China.; 6 Davis Heart & Lung Research Institute, Division of Cardiovascular Medicine, The Ohio State University College of Medicine, OH 43210, USA.

**Keywords:** mtDNA, cGAS, iNOS, Sterile inflammation, Cardiac dysfunction

## Abstract

**Background:** Sterile inflammation contributes to the pathogenesis of cardiac dysfunction caused by various conditions including pressure overload in hypertension. Mitochondrial DNA (mtDNA) released from damaged mitochondria has been implicated in cardiac inflammation. However, the upstream mechanisms governing mtDNA release and how mtDNA activates sterile inflammation in pressure-overloaded hearts remain largely unknown. Here, we investigated the role of inducible NO synthase (iNOS) on pressure overload-induced cytosolic accumulation of mtDNA and whether mtDNA activated inflammation through the cyclic GMP-AMP synthase (cGAS)-stimulator of interferon genes (STING) pathway.

**Methods:** To investigate whether the cGAS-STING cascade was involved in sterile inflammation and cardiac dysfunction upon pressure overload, cardiomyocyte-specific STING depletion mice and mice injected with adeno-associated virus-9 (AAV-9) to suppress the cGAS-STING cascade in the heart were subjected to transverse aortic constriction (TAC). iNOS null mice were used to determine the role of iNOS in cGAS-STING pathway activation in pressure-stressed hearts.

**Results:** iNOS knockout abrogated mtDNA release and alleviated cardiac sterile inflammation resulting in improved cardiac function. Conversely, activating the cGAS-STING pathway blunted the protective effects of iNOS knockout. Moreover, iNOS activated the cGAS-STING pathway in isolated myocytes and this was prevented by depleting cytosolic mtDNA. In addition, disruption of the cGAS-STING pathway suppressed inflammatory cytokine transcription and modulated M1/M2 macrophage polarization, and thus mitigated cardiac remodeling and improved heart function. Finally, increased iNOS expression along with cytosolic mtDNA accumulation and cGAS-STING activation were also seen in human hypertensive hearts.

**Conclusion:** Our findings demonstrate that mtDNA is released into the cytosol and triggers sterile inflammation through the cGAS-STING pathway leading to cardiac dysfunction after pressure overload. iNOS controls mtDNA release and subsequent cGAS activation in pressure-stressed hearts.

## Background

Cardiac dysfunction is a major health problem and the leading cause of death worldwide 1. Novel treatments are required to prevent the progression of cardiac dysfunction. Cardiac remodeling, which is characterized by interstitial fibrosis and cardiomyocyte hypertrophy, is a dangerous development leading to the failure of pump function in pressure overload-stressed hearts 2. Recently, various lines of evidence have indicated that the immune system plays an important role in the pathogenesis of cardiac fibrosis and heart failure 3,4. Both laboratory and clinical studies have shown that despite an absence of microorganism-mediated infection, chronic sterile inflammation is closely associated with the development of cardiac remodeling 5. However, our understanding on how sterile inflammation is initiated amid cardiac remodeling remains incomplete.

Inducible nitric oxide synthase (iNOS) is an important pro-inflammatory and nitrative stress mediator 6,7. The normally undetectable iNOS isoform is found to be present at high levels in cardiomyocytes in cardiac hypertrophy and heart failure 8. An abundance of NO as well as superoxide generated from the high-output iNOS results in nitrative stress in various intracellular organelles and aggravating cardiac remodeling and dysfunction 9,10; However, whether iNOS participates in the onset of sterile inflammation in pressure overload-stressed hearts remains poorly understood.

Mitochondria exert important roles in crucial cellular processes including energy production, calcium homeostasis and innate immunity 11,12. Previous studies, including from our lab, have demonstrated that pressure overload damages the mitochondria in cardiomyocytes 13,14. It has been reported that mitochondrial DNA (mtDNA) which escaped from injured mitochondria is released into the cytosol and this provokes inflammation, acting as a danger-associated molecular patterns (DAMPs) 15. As the heart possesses an abundance of mitochondria due to its high ATP demand, mtDNA may be a significant source of DAMPs that initiate sterile inflammation in diseased hearts. Considering that a high degree of nitrative stress damages the integrity of the mitochondrial membrane 16. This could result in mtDNA being released into the cytosol. Thus, iNOS may be involved in cytosolic mtDNA accumulation as well as the subsequent sterile inflammation in hearts.

The cyclic GMP-AMP synthase (cGAS)-stimulator of interferon genes (STING) pathway has recently been identified as a pattern recognition receptor (PRR), which functions as a cytosolic double-stranded DNA (dsDNA) sensor during viral or bacterial infection 17,18. Similar to bacterial DNA, mtDNA can also be recognized by the cGAS-STING pathway 19. Upon binding to mtDNA, cGAS acts through its downstream target STING. STING then induces interferon regulatory factor 3 (IRF3) phosphorylation. Phospho-IRF3 promotes the transcription of IFN and IFN-stimulated nuclear gene products leading to sterile inflammatory responses 20,21. Sterile inflammatory responses are similar to those that occur during classical infections. They both result in the recruitment of macrophages and production of inflammatory cytokines 22. Recently, it has been reported that cGAS can function as a PRR in initiating a sterile immune response in stressed hearts 23,24. Although the cGAS-STING pathway has been shown to participate in cardiac injury in diseases 25, important question remains regarding how the cGAS-STING pathway is activated in stressed hearts. Specifically, whether mtDNA activates cardiac cGAS and the associated inflammatory cascade in pressure overload-stressed hearts is unknown.

In the present study, we measured the levels of mtDNA released in the cytosol in pressure-overloaded hearts and investigated whether mtDNA triggers inflammation through the cGAS-STING pathway leading to cardiac injury. We also explored the role of iNOS in controlling mtDNA release and cGAS activation in pressure-stressed heart.

## Methods

### Animal model

C57/B6 male mice were obtained from the Experimental Animal Center of Chongqing Medical university and iNOS null (iNOS^-/-^) mice with a C57/B6 background were purchased from the Jackson Laboratory. STING conditional knockout mice were purchased from Shanghai Biomodel Organism Science & Technology Development Co., Ktd. STING^flox/flox^ mice were crossbred with Myh6-Cre mice to construct a cardiomyocyte-specific STING depletion mice (STING cKO mice). Primers: 5'-CTGCTGGTGAGTGACTTTTTGAAC-3' and 5'-ATGGGCTACTCTTGGATACACCTC-3'were used to identify the STING flox target region. Homozygous mice showed one band with 729 bp, and wildtype mice showed one band with 624 bp; Primers: 5'-TCTATTGCACACAGCAATCCA-3'and 5'-CCAGCATTGTGAGAACAAGG-3'were used for Myh6-cre PCR identification. Wildtype mice showed no band and mice with targeted showed a band with 300bp. All animal experiments were performed in accordance with the National Institutes of Health Guidelines for the Use of Laboratory Animals. The institutional Animal Care and Use Committee of Chongqing Medical University approved all the study protocols. The number of mice included in each study is indicated in the figures or the associated figure legends. Transverse aortic constriction (TAC) was performed on 8-week-old wildtype or iNOS^-/-^ mice to induce pressure overload as described previously with some modifications 14. Anesthesia was induced by 4% isoflurane for 1-2 min and then maintained with 1-1.5% isoflurane in pure oxygen until the operation was completed. The aorta was approached using bespoke tools, and a 27-gauge needle was placed next to the transverse thoracic aorta followed by ligation with a 7-0 ligature. Mice in the Sham control group underwent the same surgical procedure but without ligation. To activate the cGAS-STING pathway in iNOS^-/-^ mice, STING agonist diABZI 26 (Compound 3; Selleck Chemicals, Cat.#:S8796) was dissolved in DMSO to a final concentration of 50 mg/ml. A total of 1 mg/kg STING agonist Compound 3 was diluted in saline solution and administered to mice by intravenous injection twice a week, the dose of Compound 3 are based previous published paper 26 and our preliminary experiments (Figure S1). To test the effects of the cGAS-STING pathway in heart failure mice, serotype 9 adeno-associated viruses (AAV) were used for cGAS and STING knockdown in hearts. The AAV coding murine shRNA-cGAS and murine shRNA-STING were purchased from Hanheng Biotechnology. 4-week-old mice were chosen to receive a single bolus tail vein injection of AAV9 at 1×10^11^ viral genomes per mouse. TAC surgery was performed after 4 weeks of infection.

### Echocardiography

Echocardiography was used to assess cardiac function before and after TAC surgery using a VINNO ultrasound system for small animals. Mice were anesthetized using 4% isoflurane in 100% oxygen for induction and anesthesia was maintained using 1-1.5% isoflurane in 100% oxygen. Chest fur was removed using a chemical hair remover, then the M-mode and 2D measurements were performed. And LVEF (left ventricular ejection fraction), LEFS (left ventricular fraction shortening), LVIDd (end-diastolic left ventricular internal diameter), LVIDs (end-systolic left ventricular internal diameter), IVSd (end-diastolic interventricular septal thickness), IVSs (end-systolic interventricular septal thickness), LVPWd (end-diastolic left ventricular posterior wall thickness) and LVPWs (end-systolic left ventricular posterior wall thickness) were measured.

### Bioinformatics analysis

To investigate the expression of iNOS in different cell types, we downloaded a single nuclei RNA sequencing (snRNA-seq) datasets from the Gene Expression Omnibus database (https://www.ncbi.nlm.nih.gov/geo/query/acc.cgi?acc=GSE120064). That dataset includes the snRNA-seq data on the TAC mouse heart tissues after 0, 2, 5, 8 and 11 weeks of the TAC procedure. Seurat 27 was used for data dimensionality reduction, clustering and annotation, the annotation of cells is consistent with that of Zongna Ren *et al.* 28. Moreover, Gene Set Enrichment Analysis (GSEA) was performed using ClusterProfiler 29 and GSEABase.

### Histology

Mice were anesthetized with 5% isoflurane, and hearts were excised 3-4 weeks after TAC surgery. The death of mice was confirmed by lack of breathing and lack of a heartbeat. Before fixing with 4% paraformaldehyde, the heart was perfused with 10 mL cold PBS. Then, the heart was embedded in OCT compound; 7-μm-thick sections were used for staining. Hematoxylin and eosin staining were performed using a Hematoxylin-eosin Staining Kit (Beijing Solarbio Science & Technology Co., Ltd., Cat. #: G1121) to observe the global changes in heart size. After incubation with hematoxylin solution, sections were washed and differentiated, followed by incubation in eosin solution, dehydrated in a series of ethanol solutions (75%-100%), then cleared in xylene and mounted in neutral gum. Masson's trichrome was used to assess collagen density using a Masson's Trichrome Staining Kit (Beijing Solarbio Science & Technology Co., Ltd. Cat. #: G1346) according to the manufacturer's instructions. To evaluate the cardiomyocyte cross-sectional area, FITC labeled Wheat Germ Agglutinin and Alexa Fluor 647 labeled WGA (Thermo Fisher Scientific, Cat. #: W834 and W32466) were used according to the manufacturer's instructions.

### Immunohistochemistry

Paraffin sections form human hearts were obtained from the Department of Forensic Medicine, College of Basic Medicine, Chongqing Medical University. All paraffin embedded sections were the remnants of heart tissue used for medical forensic examinations from donors with hypertensive heart disease. Participants or their patients' family members were informed, and they provided consent prior to the inclusion of these samples in the study. Additionally, the experiments conformed to the principles outlined in the Declaration of Helsinki. Sections were incubated in 0.2% Triton X for 10 min at room temperature, and 10% goat serum in PBS was used to block non-specific antibody sites. Then, sections were incubated with primary antibodies against iNOS (Wuhan Servicebio Technology Co., Ltd., 1:200, Cat. #: GB11119), cGAS (Santa Cruz Biotechnology, Inc., 1:50, Cat. #: SC-515777), STING (Cell Signaling Technology, Inc., 1:200, Cat. #: 13647) or phospho-interferon regulatory factor 3 (IRF3) (Ser396) (Affinity Biosciences, Ltd., 1:100, Cat. #: AF2436) in PBS with 10% goat serum overnight at 4 ℃. After washing with PBS, sections were incubated with the corresponding HRP-conjugated secondary antibodies (Thermo Fisher Scientific, Inc., Cat #: 31460 and 31430) for 1 h at room temperature, and signals were developed using a DAB Substrate Kit (Wuhan Servicebio Technology Co., Ltd., Cat. #: G1212). Images were taken using a microscope (DM4 B, Leica Microsystems, Inc.).

### RNA isolation and quantitative PCR

Total RNA was isolated using TRIzol^®^ (Invitrogen; Thermo Fisher Scientific, Inc., Cat. #: 15596026) according to the manufactures' protocol. A total of 1 μg RNA was used for reverse transcription using a high‑capacity cDNA Synthesis Kit (Takara Bio, Inc., Cat. #: RR064B) according to the manufacturer's instructions. Quantitative PCR was performed using SYBR Green. The complete list of primers and their sequences are shown in Table S1.

### Detection of mtDNA content in the cytosol

DNA was extracted from the cytosol as described previously 30. Briefly, 20 μg heart tissue was homogenized in 500 μL buffer containing 150 mM NaCl, 50 mM HEPES (pH 7.4), and 25 μg/mL digitonin. The homogenates were spun on a roller mixer for 10 min then centrifuged three times at 980 x g for 5 min. The cytosolic supernatants were collected and centrifuged at 17000 x g for 25 min to obtain the cytosolic fraction without mitochondrial and nuclear contaminants, which was then used for DNA extraction using a QIAquick Nucleotide Removal Columns (Qiagen, GmbH, Cat. #: 28306). Total DNA was extracted from another 10 μg of heart tissues and was used as the control for cytosolic mtDNA. Then, the total DNA and cytosolic DNA were used for quantitative PCR. No nuclear DNA in the cytosolic fractions was detected, suggesting nuclear lysis did not occur.

### Immunofluorescence

Co-localization of dsDNA and mitochondria was used to visualize mtDNA in the cytosol. Sections were incubated in 0.2% Triton X for 10 min at room temperature, and 10% goat serum in PBS was used to block non-specific binding. Then, sections were incubated with a primary antibody against dsDNA (Abcam, 1:100, Cat. #: ab27156) and Tom20 (Abcam, 1:100, Cat. #: ab186735) overnight at 4 ℃, followed by a FITC-conjugated or Fluor594-conjugated secondary antibody for 1 h at room temperature in the dark. Next, sections were viewed using a Laser Scanning Confocal Microscope (Leica Microsystems, Inc., USA).

### Flow cytometry and cell sorting

Mice were treated with heparin and then anesthetized with 4% isoflurane for 1-2 min for induction, which was maintained with 3% isoflurane. The hearts were excised and perfused with cold PBS for 10 min. Tissues from the left ventricle were minced and digested using Accumax (Stemcell Technologies, Cat. #: 07921) for 1 h at 37 ℃. Then the tissues were blown with a dropper and passed through a 40 μm strainer. After centrifugation at 100 rpm for 20 min followed by Percoll gradient centrifugation, cells were incubated with CD45.2-Alexa Fluor 700 (1:100, Cat. #: 560693), F4/8-Alexa Fluor 647 (1:25, Cat. #: 565854), Ly6c-BV421 (1:50, Cat. #: 562727), Ly6G-APC-CyTN7 (1:100, Cat. #: 560606) (all from BD Biosciences), CD11b-PerCP-Cyanine5.5 (1:25, Cat. #: 45-0112-82) and CD206-PE (1:50, Cat. #: 141706) (both from BioLegend, Inc.) for 30 min at room temperature in the dark. Next, cells were washed and resuspended in FACS scan buffer and flow cytometry was performed on a CytoFLEx platform (Beckman Coulter, Inc.) and data were analyzed using CytExpert. Total macrophage/monocyte population was represented by the CD45.2^+^Ly6G^-^CD11b^+^F4/80^+^ cells. Then, in this cell population, CD45.2^+^Ly6G^-^CD11b^+^F4/80^+^CD206^high^ cells were considered to represent the reparative M2 macrophages, and the CD45.2^+^Ly6G^-^CD11b^+^F4/80^+^CD206^low^ cells were considered to represent the inflammatory M1 macrophages as described previously 24.

### Neonatal rat ventricular myocytes culture and transfection

Neonatal rat ventricular myocytes were isolated from 1-3-day-old neonatal pups as described previously^14^. Cells were seeded in 6-well flasks and maintained in M199 medium with 10% FBS at 37 ℃ in a humidified incubator supplied with 5% CO_2_. The murine iNOS gene (Stratagene; Agilent Technologies, Inc.) was constructed as described previously 31. Transfection of the iNOS plasmid or siRNA-STING was performed using jetOPTIMUS (Polyplus-transfection SA, Cat. #: 101000051) according to the manufactures' instructions. After 48 h, transfected cells were harvested for the subsequent experiments.

mtDNA was isolated from frozen heart tissues using a commercialized mtDNA Extractor (FUJIFILM Wako Pure Chemical Corporation, Cat. #: 291-55301) according to the manufacturer's instructions. mtDNA solution was stored at 4 ℃ with 1 mM EDTA and used within 2 weeks. A total of 2.5 μg/mL mtDNA was transfected using Lipo3000 (Invitrogen; Thermo Fisher Scientific, Inc., Cat. #: L3000015). Cell lysates were harvested for subsequent measurement after 24 h.

### mtDNA depletion in H9C2 cells

H9C2 cells were used to generate mtDNA-depleted cells. Briefly, cells were cultured in DMEM supplemented with 10% FBS in a humidified incubator at 37 °C supplied with 5% CO_2_. For mtDNA depletion, cells were cultured with 100 ng/mL ethidium bromide (EtBr) for 3 days, and after 3 passages, mtDNA content was evaluated using quantitative PCR as described above.

### Western blotting

The total protein content of heart tissues was extracted using a Column Tissue and Cell Protein Extraction Kit (EpiZyme, Shanghai, Cat #: PC201plus). The protein concentration was measured using a BCA assay and samples were incubated using Blue Loading Buffer for 30 min at 60 ℃. Equivalent amounts of samples were loaded onto an SDS-PAGE gel and then transferred to PVDF membranes (for iNOS detection, nitrocellulose membranes were used). After blocking with 5% milk in TBS with 0.1% Tween 20 for 60-90 min at room temperature, the immunoblots were incubated with the specific primary antibodies overnight at 4 ℃, and then with secondary antibodies for 60 min at room temperature. Signals were visualized using ECL-plus reagent, and images were obtained using the Bio-Rad system (Bio-Rad Laboratories, Inc.). The primary antibodies used for western blotting were: Anti‑cGAS (1:1,000, Abclonal, Cat. #: A8335), anti‑STING (1:1,000, Cell Signaling Technology, Inc., Cat. #: 13647), anti‑IRF3 (1:1,000, Cell Signaling Technology, Inc., Cat. #: 4302), anti‑phospho‑IRF3 (1:500, Cell Signaling Technology, Inc., Cat. #: 13647), anti‑iNOS (1:500, Abcam, Cat. #: ab15323) and anti‑GAPDH (1:5,000; ProteinTech Group, Inc., Cat. #: ab10494).

### Statistical analysis

All statistical analyses were performed using GraphPad Prism version 7 (GraphPad Software, Inc.). All data were presented as the mean ± SEM. Data were compared using an unpaired Student's t-test or a one-way or two-way ANOVA followed by a Tukey's multiple-comparison post-hoc test. P < 0.05 was considered to indicate a statistically significant difference.

## Results

### iNOS contributes to cytosolic mtDNA accumulation and cGAS activation in pressure overload-stressed heart

**Figure 1:**
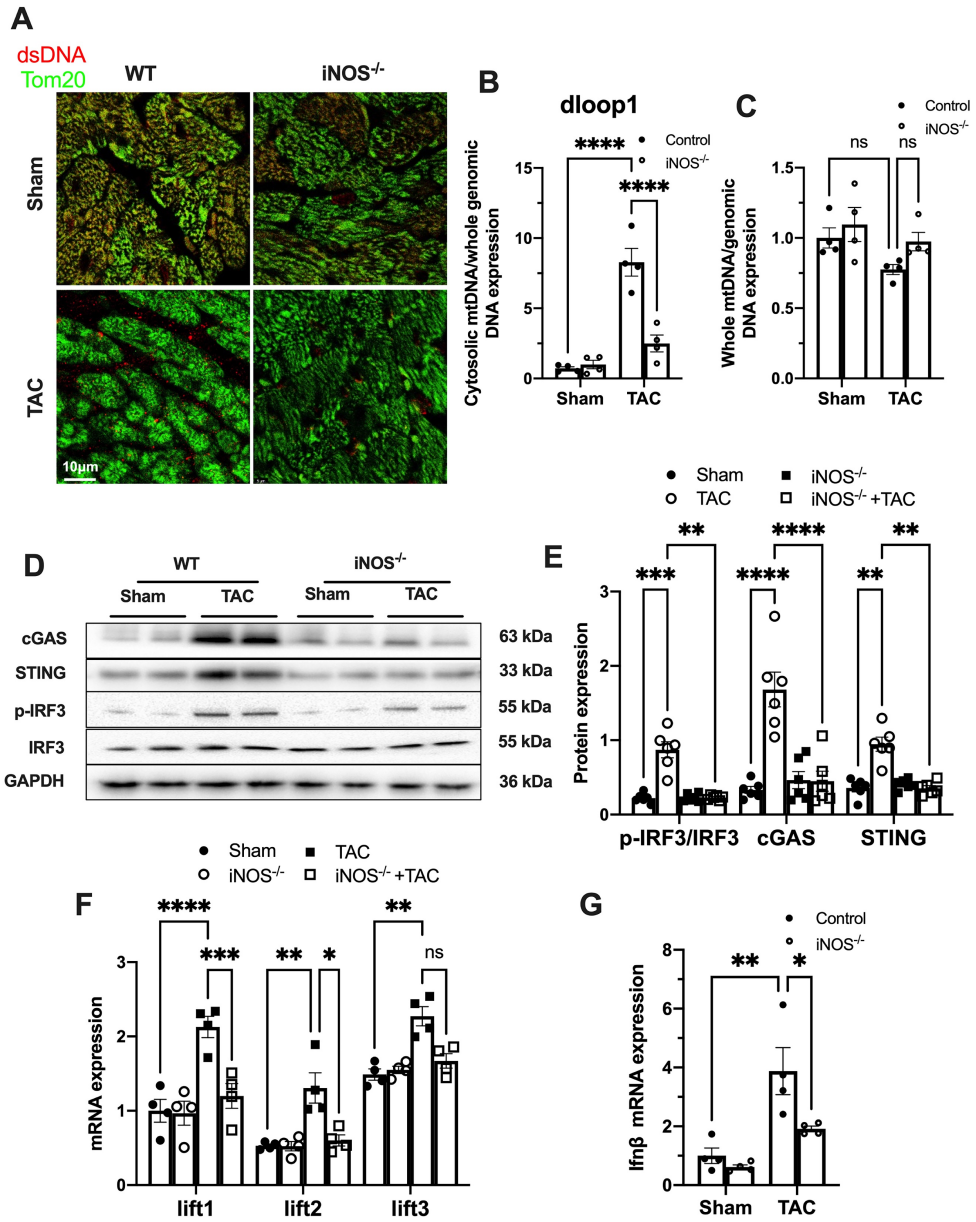
** iNOS contributes to cytosolic mtDNA accumulation and cGAS activation in pressure overload-stressed heart. (A)** Representative co-immunostaining of dsDNA and mitochondria (Tom20) showing cytosolic DNA in heart tissues.** (B)** Cytosolic mtDNA content in the hearts of TAC mice were measured using quantitative-PCR, n = 4. **(C)** Total mtDNA in the hearts of TAC mice, n = 4.** (D-E)** iNOS deficiency decreased cGAS-STING expression and IRF-3 phosphorylation in the hearts of TAC mice, n = 6. **(F-G)** iNOS deficiency reduced the mRNA expression levels of IFN-β and ISG activated by cGAS-STING in TAC hearts, n = 4. Data are presented as the mean ± SEM. Statistical analysis was performed using a 1 or 2-way ANOVA with a Tukey's multiple-comparison post-hoc test comparisons between multiple groups. TAC, transverse aortic constriction; iNOS. inducible NO synthase; mtDNA, mitochondrial DNA; dsDNA, double-stranded DNA; cGAS, cyclic GMP-AMP synthase; STING, stimulator of interferon genes; IFN-β, interferon-β. *, *P* < 0.05. **, *P* < 0.01.***, *P* < 0.001. ****, *P* < 0.0001.

While iNOS was not detectable in normal heart, a significant increase in iNOS expression was measured in the pressure-stressed heart after 1 week and peaked at 3 weeks after the TAC surgery (Figure S2A-B). The single-cell sequencing results showed that iNOS expression mainly increased in cardiomyocytes (Figure S2C), but not in endothelial cells, fibroblasts, or even in macrophages (Figure S2D-F). Moreover, gene set enrichment analysis indicated that the differentially expressed genes were primarily associated with cytosolic DNA sensing pathway (Figure S2G). We thus investigated whether iNOS was involved in the leakage of mtDNA into the cytosol and cGAS-STING pathway activation using iNOS^-/-^ mice. Indeed, iNOS deficiency significantly reduced the cytosolic mtDNA content in the heart tissue from the TAC mice compared with that of the sham group (Figure 1A-B). However, total amount of mtDNA in TAC heart was not significantly affected by iNOS knockout (Figure 1C). Moreover, iNOS knockout blunted cGAS-STING activation in the TAC mice heart, as evidenced by decreased expression of members of the cGAS-STING pathway (Figure 1D-E), the transcript levels of IFN-β and IFN-stimulated nuclear genes (ISGs) (Figure 1F-G). These data showed that iNOS served as an upstream regulatory molecule governing cGAS-STING activation in TAC heart.

### iNOS-induced mtDNA release activates the cGAS-STING pathway

**Figure 2:**
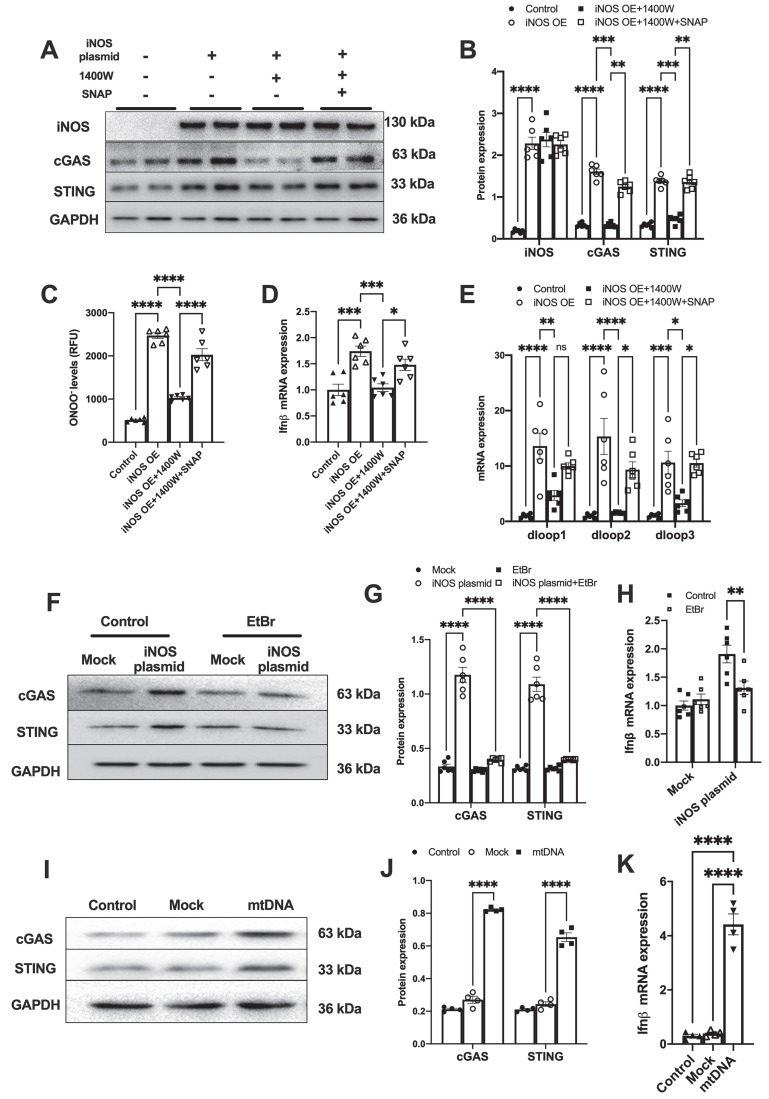
** iNOS-induced mtDNA release activates the cGAS-STING pathway. (A-B)** The expression of cGAS and STING in cells transfected with iNOS, n = 6. **(C)** The ONNO levels were measured to reflect nitrative stress, n = 6.** (D)** The mRNA expression levels of IFN-β were measured, n = 6. **(E)** iNOS expression increased the cytosolic mtDNA content in cardiomyocytes, n = 6.** (F-H)** mtDNA depletion blunted the effects of iNOS on increasing cGAS-STING and IFN-β expression, n = 6.** (I-K)** mtDNA increased cGAS-STING and IFN-β **(N)** expression in isolated cardiomyocytes. Data are presented as the mean ± SEM. Statistical analysis was performed using a 1 or 2-way ANOVA with a Tukey's multiple-comparison post-hoc test comparisons between multiple groups. iNOS. inducible NO synthase; SNAP, S-Nitroso-N-acetyl-DL-penicillamine; EtBr, ethidium bromide; IFN-β, interferon-β. *, *P* < 0.05. **, *P* < 0.01.***, *P* < 0.001. ****, *P* < 0.0001.

To further demonstrate that iNOS expression leads to mtDNA leakage into the cytosol, we transfected a plasmid encoding iNOS into the isolated cardiomyocytes (Figure S3A). iNOS directly increased ONOO^-^ content and cGAS-STING activation level in isolated cardiomyocytes, and these effects could be blunted by iNOS inhibition (Figure 2A-D). Addition of the NO donor S-Nitroso-N-acetyl-DL-penicillamine (SNAP) induced nitrative stress and rescued the effects of iNOS in inhibitor-treated cardiomyocytes (Figure 2A-D). Moreover, iNOS expression markedly increased the mtDNA content in the cytosol of isolated cardiomyocytes, which could be recused by the selective iNOS inhibitor 1400W (Figure 2E). However, adding the NO donor SNAP reversed the effects of iNOS inhibition on mtDNA leakage in cardiomyocytes (Figure 2E). These data obtained from cultured cardiomyocytes agreed with those obtained from the *in vivo* experiments on mice. They collectively demonstrated that iNOS-derived NO and ONOO^-^ were responsible for cytosolic mtDNA accumulation and cGAS pathway activation in pressure overload-stressed heart.

To explore whether iNOS modulated cGAS activation through mtDNA, we generated mtDNA-depleted H9C2 cells using EtBr (Figure S3B). Our results showed that the iNOS-induced increase in cGAS and STING expression was almost totally blunted by mtDNA depletion (Figure 2F-G). In agreement with this effect, transcript levels of IFN-β were also significantly decreased by mtDNA depletion (Figure 2H). To directly demonstrate that mtDNA activated cGAS, we transfected mtDNA into isolated cardiomyocytes. As expected, increased cGAS-STING expression and IFN-β transcript levels were seen in mtDNA-transfected cells (Figure 2I-K). These data demonstrated that mtDNA was a ligand of cGAS and iNOS could activate the cGAS-STING pathway by increasing the cytosolic mtDNA content.

### iNOS deficiency alleviates pressure overload-induced sterile inflammation and cardiac injury

**Figure 3:**
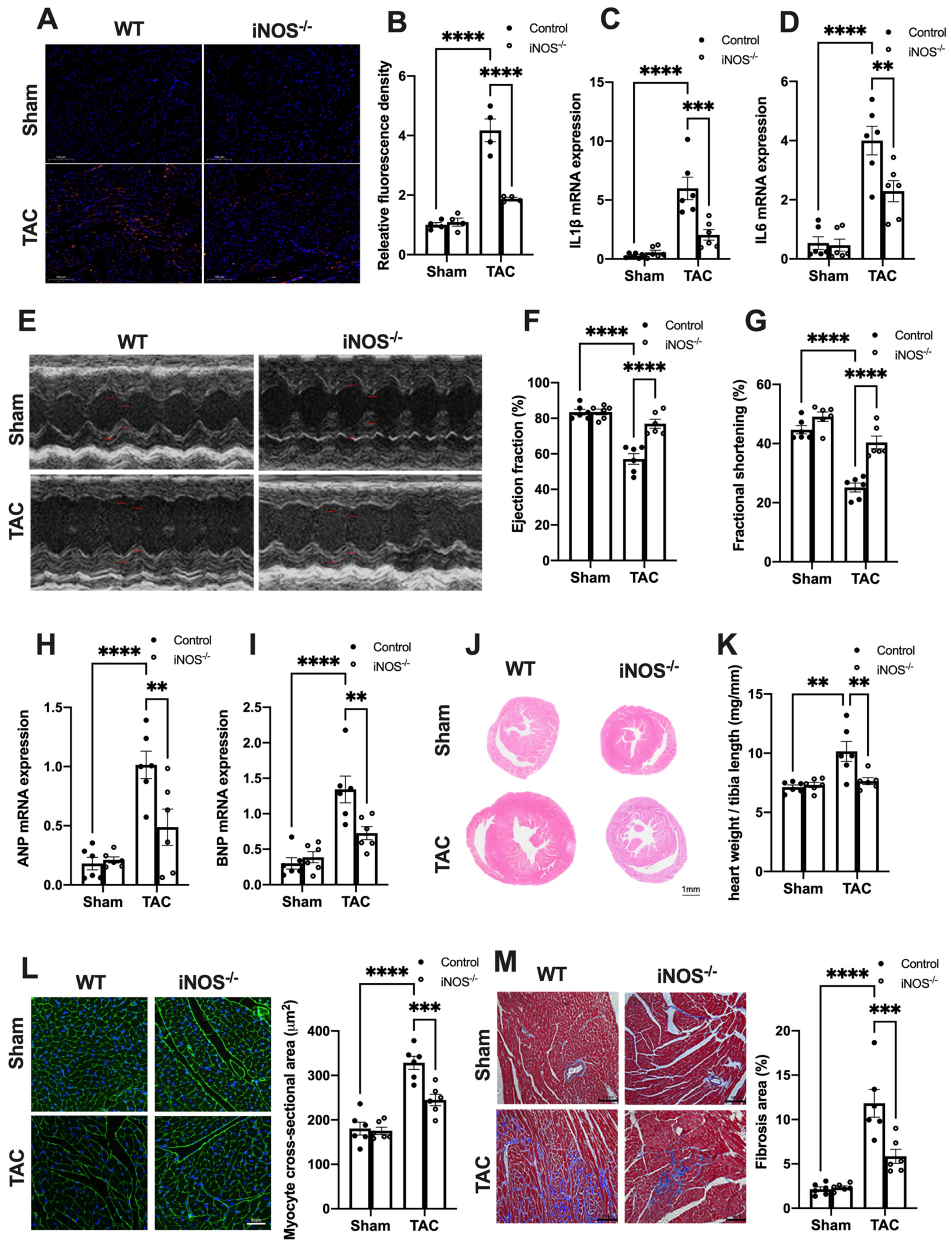
** iNOS deficiency alleviates pressure overload-induced sterile inflammation and cardiac injury. (A-D)** iNOS deficiency decreased the content of M1 macrophages and transcript levels of IL-1β, IL-6 in TAC hearts, n = 6.** (E-G)** iNOS deficiency improved cardiac function. Representative images of echocardiograms are shown in **(E)**. LV ejection fraction and fractional shortening are shown in **(F)**and **(G)** respectively, n = 6. **(H-I)** Deficiency of iNOS reduced the mRNA expression levels of ANP and BNP induced by TAC, n = 6. **(J)** Representative hematoxylin-eosin staining of myocardial tissues from WT or iNOS^-/-^ mice after TAC. Scale bar: 1 mm. The HW/TL ratio are shown in **(K)**, n = 6. **(L)** Representative WGA staining of midventricular sections to assess hypertrophy of cardiac myocytes. Scale bar: 100 μm. Quantitative analysis of WGA staining is shown in right, n = 6. **(M)** Representative images of Masson staining of the heart sections. Scale bar: 100 μm. The results of the statistical analyses are shown in right, n = 6. Data are presented as the mean ± SEM. Data were analyzed using a two-way ANOVA with a Tukey's multiple-comparison post-hoc test. TAC, transverse aortic constriction; WT, wildtype; HW/TL, heart weight and tibia length; WGA, wheat germ agglutinin; LV, left ventricular; IL, interleukin; ANP, atrial natriuretic peptide; BNP, brain natriuretic peptide. *, *P* < 0.05. **, *P* < 0.01.***, *P* < 0.001. ****, *P* < 0.0001.

We next evaluated the effects of iNOS on inflammation and cardiac injury in TAC mice. iNOS deficiency led to a significant reduction in the content of CD68^+^ M1 macrophages and expression of transcripts encoding for proinflammatory proteins (Figure 3A-D). Moreover, the impaired pump function was also improved by iNOS deficiency, as evidenced by the increase in left ventricular (LV) ejection fraction (EF) and fractional shortening (FS) (Figure 3E-G), as well as the reduced ANP and BNP levels in the TAC heart (Figure 3H-I). The iNOS deficient TAC mice exhibited attenuated cardiac remodeling (Figure 3J-L) and myocardial fibrosis (Figure 3M). Together, these data showed that iNOS deficiency prevented cGAS-STING-induced sterile inflammation and thus protected the cardiac function of TAC mice.

### cGAS-STING pathway activation blunts the protective role of iNOS deficiency on TAC heart

**Figure 4:**
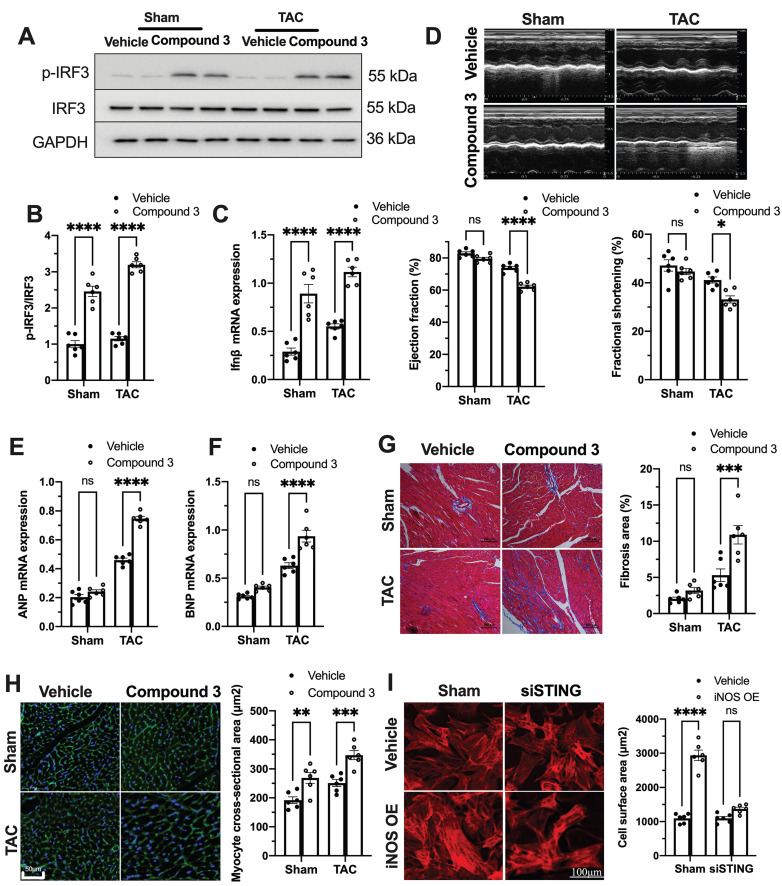
** cGAS-STING pathway activation blunts the protective role of iNOS on TAC heart. (A-C)** STING agonist Compound 3 increased IRF-3 phosphorylation and IFN-β expression in the heart of iNOS^-/-^ mice, suggesting the activation of the cGAS-STING pathway, n = 6. **(D)** STING agonist Compound 3 aggravated cardiac function in iNOS^-/-^ TAC mice as shown in the representative echocardiograms. LV ejection fraction and fractional shortening are shown below, n = 6.** (E-F)** Compound 3 increased the mRNA expression levels of ANP and BNP induced by TAC in iNOS^-/-^ mice, n = 6. **(G)** Representative images of Masson staining of the heart sections. Scale bar: 100 μm. Results of statistical analysis shown in right, n = 6. **(H)** Representative WGA staining to assess hypertrophy of cardiac myocytes. Scale bar: 50 μm. Quantitative analysis of WGA staining was shown in right, n = 6. **(I)** Representative phalloidine staining of isolated cardiomyocytes. Scale bar: 100 μm. Quantitative analysis was shown in right, n = 6. Data were presented as the mean ± SEM and were analyzed using a two-way ANOVA with a post-hoc Tukey's multiple-comparison test. TAC, transverse aortic constriction; WT, wildtype; WGA, wheat germ agglutinin; ANP, atrial natriuretic peptide; BNP, brain natriuretic peptide. *, *P* < 0.05. **, *P* < 0.01.***, *P* < 0.001. ****, *P* < 0.0001.

To further establish the role of the cGAS-STING pathway as a downstream mechanism of iNOS, iNOS null mice were administrated the STING agonist Compound 3 and subjected to TAC surgery. STING agonist induced cGAS-STING pathway activation in the hearts of iNOS knockout mice, as evidenced by the significant increase in IRF3 phosphorylation (Figure 4A-B) and elevated IFNβ expression levels (Figure 4C). iNOS deficiency improved cardiac function as shown in Figure 3. However, STING agonist notably reduced the EF and FS in iNOS knockout TAC mice (Figure 4D). The increased ANP and BNP levels also suggested that the STING agonist aggravated cardiac function in iNOS knockout mice after TAC (Figure 4E-F). iNOS deficiency attenuated interstitial fibrosis and cardiac remodeling induced by pressure overload. However, those protective effects were also abolished by STING activation (Figure 4G-H). These results demonstrated that cGAS-STING activation reversed the protective effects of iNOS knockout on cardiac remodeling and dysfunction. In addition, we found that iNOS-induced cardiomyocyte hypertrophy was also blunted by STING knockdown *in vitro* (Figure 4I). Together, these findings indicated the crucial role of the cGAS-STING pathway in iNOS-induced cardiac injury.

### Disruption of the cGAS-STING in myocardium alleviates cardiac injury in TAC mice

**Figure 5:**
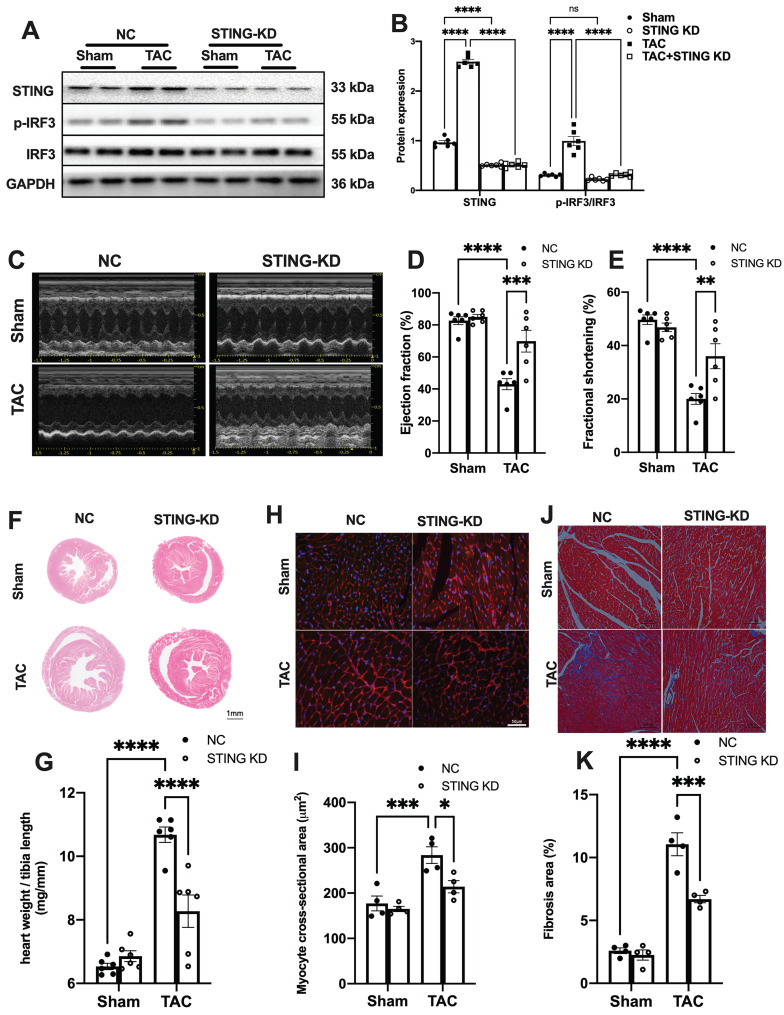
** STING knockdown reduces the expression of inflammatory markers and alleviates cardiac injury in TAC heart. (A-B)** STING knockdown suppressed the activation of the IRF-3 phosphorylation, n = 6. **(C-E)** Cardiac-specific cGAS-STING pathway disruption improved cardiac function. Representative echocardiograms are shown in **(C)**. LV ejection fraction and fractional shortening are shown in **(D) and (E),** n = 6. **(F)** Representative images of hematoxylin-eosin staining of myocardial tissue from NC or STING knockdown mice. Scale bar: 1 mm. **(G)** The HW/TL ratio, n = 6. **(H-I)** Representative images of WGA staining to assess hypertrophy of cardiac myocytes. Scale bar: 50 μm, n = 4. **(J-K)** Representative images and analysis of Masson staining of heart sections to assess fibrosis. Scale bar: 100 μm, n = 4. Data are presented as the mean ± SEM and were analyzed using a two-way ANOVA with a post-hoc Tukey's multiple-comparison test. TAC, transverse aortic constriction. NC, negative control; KD, knockdown; *, *P* < 0.05. **, *P* < 0.01.***, *P* < 0.001. ****, *P* < 0.0001.

**Figure 6:**
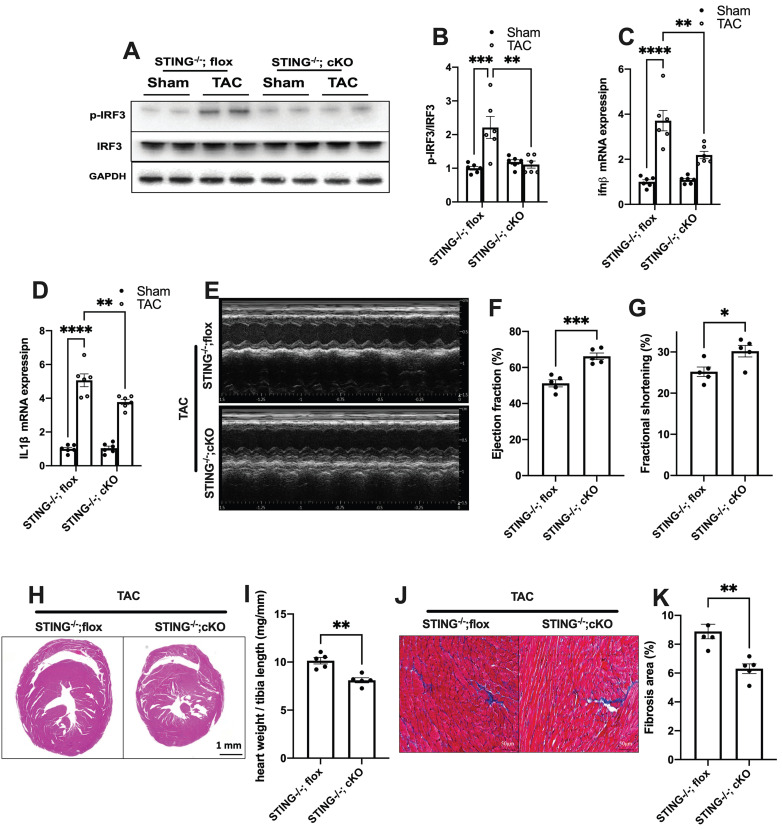
** Cardiomyocyte-specific STING deficiency alleviates cardiac injury in TAC mice. (A-C)** Cardiomyocyte-specific STING deficient suppressed the IRF-3 phosphorylation and IFN-β expression in the TAC heart, n = 6.** (D)** Quantitative PCR was used to measure the transcript levels of IL1β in TAC heart, n = 6. **(E-G)** Cardiomyocyte-specific STING deficient improved cardiac function. Representative echocardiograms are shown in **(E)**. LV ejection fraction and fractional shortening are shown in **(F) and (G),** n = 5. **(H)** Representative images of hematoxylin-eosin staining. Scale bar: 1 mm. **(I)** The HW/TL ratio, n = 5. **(J-K)** Representative images and analysis of Masson staining of heart sections to assess fibrosis. Scale bar: 100 μm, n = 5. Data were presented as the mean ± SEM and were analyzed using a two-way ANOVA with a post-hoc Tukey's multiple-comparison test. TAC, transverse aortic constriction. *, *P* < 0.05. **, *P* < 0.01.***, *P* < 0.001. ****, *P* < 0.0001.

To reconfirm the direct role of cGAS-STING pathway in mediating sterile inflammatory responses in TAC heart, we used AAV9 encoding shRNA targeting cGAS or STING to disrupt the cGAS-STING pathway in the hearts. The results showed that cGAS or STING knockdown (cGAS KD or STING KD) almost completely abrogated the TAC-induced IRF3 phosphorylation (Figure 5A-B and Figure S4A). Although the cytosolic mtDNA content was not affected (Figure S4B and Figure S5A), TAC-induced elevation of IFNβ and ISGs were largely prevented after disruption of the cGAS-STING pathway (Figure S4C-E and Figure S5B-E). The levels of inflammatory cytokines were also suppressed by cGAS or STING knockdown (Figure S4F-H and Figure S5F-H). cGAS or STING knockdown also mitigated pressure overload-induced cardiac dysfunction as evidenced by the improved LVEF and FS (Figure 5C-E, Figure S6A-C), as well as reducing ANP and BNP levels in the TAC hearts (Figure S6D-E and Figure S7). Moreover, suppression of the cGAS-STING pathway resulted in significantly less pressure overload-induced hypertrophy as evidenced by the change in morphology (Figure 6F-I and Figure S6F-I) and echocardiography (Figure S8-9). In addition, disruption of the cGAS-STING pathway also significantly reduced interstitial fibrosis in response to pressure overload (Figure 5J-K, Figure S6J-K).

To further define the role of cGAS-STING pathway activation in myocardium, we next generated a cardiomyocyte-specific STING deficient mice. STING^flox/flox^ mice were cross-bred with Myh6-Cre mice to construct a cardiomyocyte-specific STING depletion mice (STING cKO mice) (Figure S10A-C). The result showed that STING cKO mice showed an 80% reduction of STING protein expression, suggesting that cardiomyocyte expresses STING at high level (Figure S10D-E). Similar to disruption of the cGAS-STING pathway in the heart, depleting STING expression in cardiomyocytes also eliminated the TAC-induced IRF3 phosphorylation and inflammation in TAC heart (Figure 6A-D). STING cKO mice also showed improved cardiac pump function as evidenced by the improved LVEF and FS (Figure 6E-G), reduced cardiac remodeling and interstitial fibrosis in response to pressure overload (Figure 6H-K).

Taken together, the above results collectively indicated that the cGAS-STING pathway-mediated inflammatory responses contributed to adverse remodeling and cardiac dysfunction, and suppressing this cascade blunted these detrimental effects.

### Disruption of the cGAS-STING pathway increases M2 macrophage polarization in TAC mice

**Figure 7:**
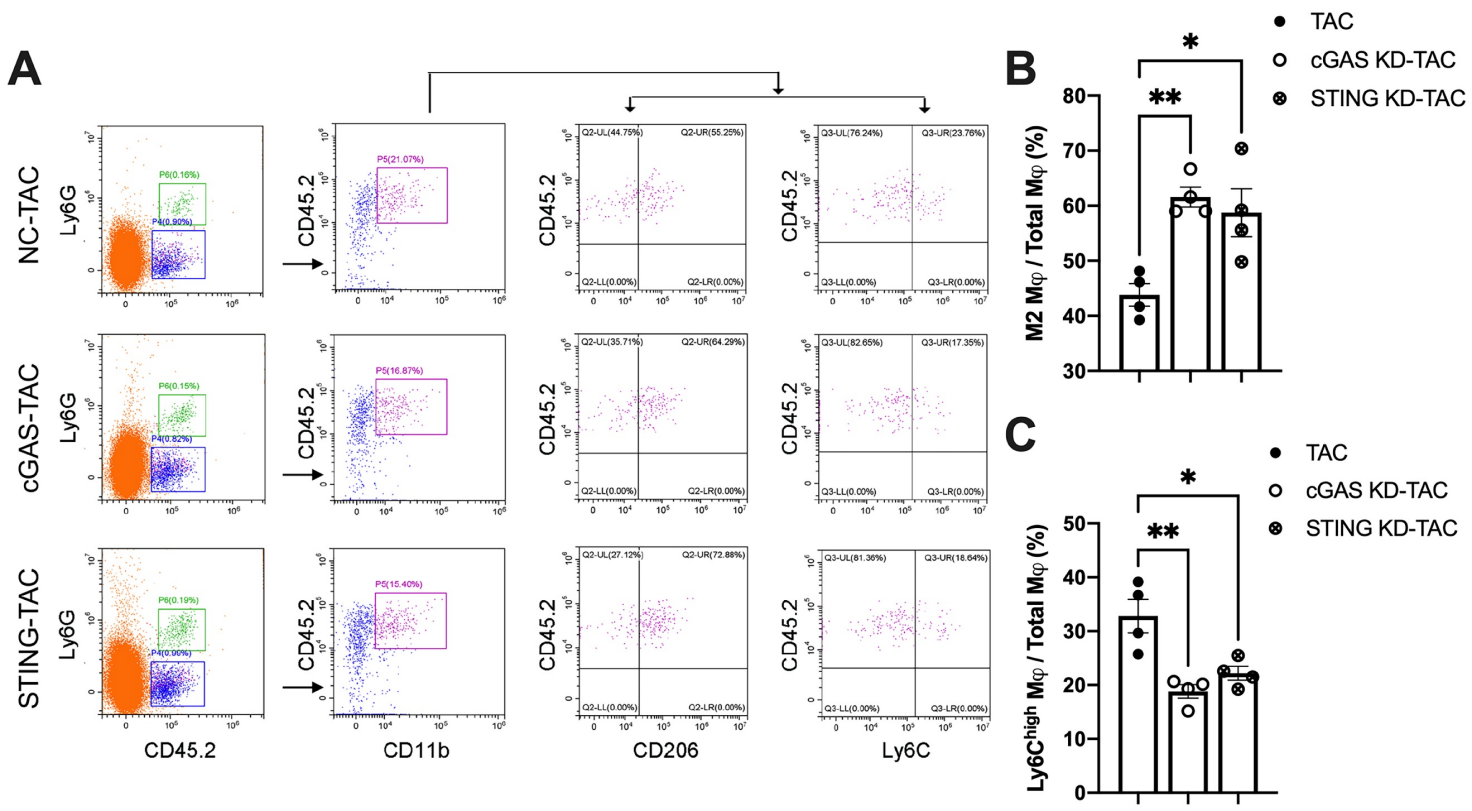
** Disruption of the cGAS-STING pathway increases the proportion of M2 macrophages and alleviates cardiac injury in TAC mice. (A)** Flow cytometry analysis of macrophages from the hearts of NC, cGAS KD and STING KD mice after TAC. **(B-C)** Percentage of M1 or M2 macrophages in the TAC heart, n = 4. Data are presented as the mean ± SEM and were analyzed using a one-way ANOVA with a Tukey's multiple-comparison post-hoc test. TAC, transverse aortic constriction. NC, negative control; KD, knockdown; *, *P* < 0.05. **, *P* < 0.01.

Macrophages infiltration into the heart is an important mechanism exacerbating inflammatory responses and cardiac dysfunction 32-34. As shown in Figure 7A, cGAS or STING KD reduced the counts of CD45.2^+^Ly6G^-^CD11b^+^F4/80^+^CD206^low^ M1 macrophages. In contrast, the quantity of CD45.2^+^Ly6G^-^CD11b^+^F4/80^+^CD206^high^ M2 macrophages was significantly increased, indicating that macrophage polarization had occurred. Ly6C is another cell surface maker used to distinguish macrophage subtypes (Figure 7A-B). The results showed that the percentage of Ly6C^high^ macrophages was also reduced in the heart of cGAS KD or STING KD mice (Figure 7C). These two lines of evidence suggested that activation of the cGAS-STING cascade could exacerbate the recruitment of pro-inflammatory M1 macrophages. Suppressing cGAS-STING signaling may promote macrophage polarization towards the anti-inflammatory M2 subtype and mitigate cardiac inflammatory responses.

### The iNOS-mtDNA-cGAS axis is clinically relevant in human hypertensive heart tissue

**Figure 8:**
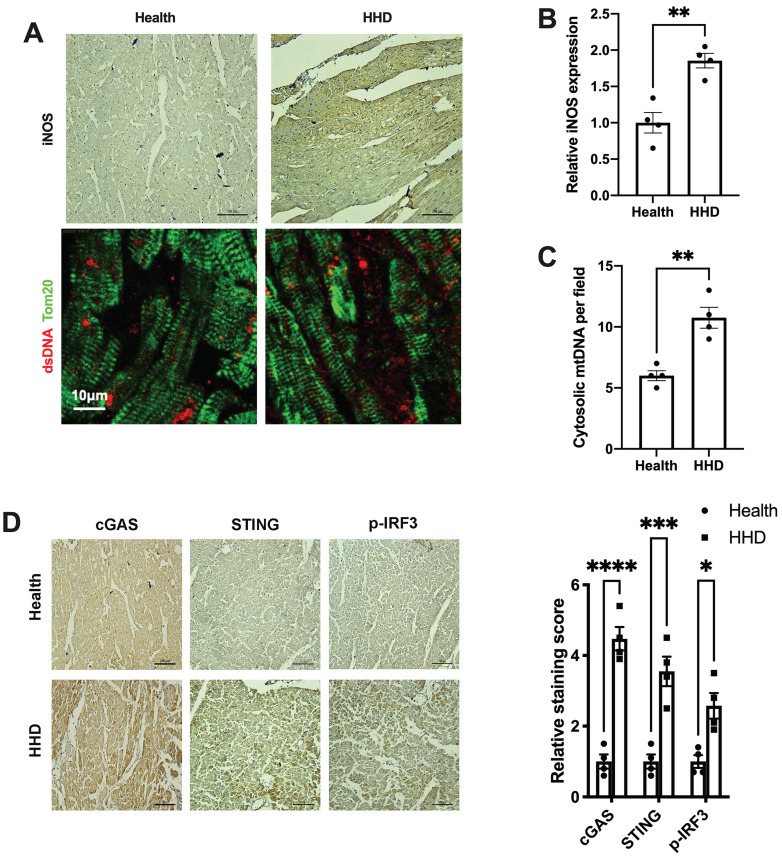
** Relevance of iNOS-mtDNA-cGAS axis in human hypertensive heart. (A)** Upper panel: Representative immunohistochemical staining of iNOS. Scale bar: 100 μm. Lower panel: Representative images of co-immunostaining of dsDNA and the mitochondria (Tom20), showing increased cytosolic DNA levels in heart tissue. Scale bar: 10 μm.** (B)** Results of the statistical analysis of iNOS expression. **(C)** Results of the statistical analysis of cytosolic mtDNA content. n = 4. **(D)** Representative images of immunohistochemical staining to assess the expression of cGAS, STING and p-IRF3 in the hypertensive heart. Scale bar: 100 μm. Results of the statistical analysis are shown in right, n = 4. Data are presented as the mean ± SEM and data were analyzed using a Student's t-test and a one-way ANOVA with a Tukey's multiple-comparison post-hoc test. HHD, hypertensive heart disease. cGAS, cyclic GMP-AMP synthase; STING, stimulator of interferon genes; iNOS. inducible NO synthase; mtDNA, mitochondrial DNA; *, *P* < 0.05. **, *P* < 0.01.***, *P* < 0.001. ****, *P* < 0.0001.

Finally, we sought to determine the implications of the above findings in human disease. We examined iNOS expression, cytosolic mtDNA content and the status of the cGAS-STING cascade in myocardial sections from individuals with or without hypertensive heart disease. Results showed that iNOS expression and cytosolic mtDNA content were markedly increased in the hypertrophic heart (Figure 8A-C). Moreover, we observed a significant increase in cGAS and STING expression in the hypertensive heart (Figure 8D). Parallel to the changes of cGAS and STING, the levels of phospho-IRF3 were also elevated (Figure 8D). These data agreed with the findings from the animal studies and underscored the concept that iNOS lead to cytosolic mtDNA accumulation resulting in activation of the cGAS-STING cascade during human cardiac remodeling and in hypertensive heart disease.

## Discussion

Growing evidence suggests that sterile inflammation is strongly associated with the adverse outcomes of various cardiovascular diseases 35. Therefore, clarifying the mechanisms that trigger sterile inflammation in diseased heart may highlight novel therapeutic targets. The present study revealed that mtDNA functions as a DAMP to initiate a sterile inflammatory response leading to cardiac damage in pressure-overload heart. In view of the abundance of mitochondria in cardiomyocytes 36, mtDNA may be of particular significance in priming inflammatory responses in heart. The findings in the present study were obtained from TAC mice or hypertensive patients, thus further investigations on the roles of mtDNA in inflammatory responses of other heart diseases are warranted.

As abundance of NO from iNOS results in nitrative stress and impairs the integrity of the mitochondrial membrane 6,16. We hypothesized that iNOS-induced nitrative stress leads to mtDNA release and then mtDNA activates cGAS-STING inflammatory pathway in TAC heart. This notion was proven in the present study using the iNOS null mice. iNOS deficiency reduces the cytosolic mtDNA content and blunts the inflammatory response in TAC heart. Conversely, iNOS expression in cells induced increased ONOO^-^ levels and promote mtDNA release into the cytosol, and these effects could be blunted by iNOS inhibition. In addition, the NO donor SNAP also increased ONOO^-^ levels and cytosolic mtDNA content in 1400W-treated cardiomyocytes, suggesting that iNOS-induced nitrative stress mediated the release of mtDNA into the cytoplasm, but not iNOS itself. These findings not only establish iNOS as a key regulator controlling mtDNA release, but also provide novel insights in to understating the detrimental effects of iNOS on stressed heart.

There is an interesting finding needed to be pointed out. We performed single-cell sequencing analysis with a public database and the results showed that iNOS expression mainly increased in cardiomyocytes, but not in endothelial cells, fibroblasts, or even in macrophages. The difference in cell types that expresses iNOS indicates that the mechanisms mediating iNOS expression is different from those in infection.

cGAS is a PRR that senses exogenous DNA from bacteria or viruses and activates the innate immune system to fight against infection 37,38. While the cGAS-STING pathway has been implicated in several types of cardiovascular injuries 39, the upstream mechanisms governing cGAS activation are not well understood. The present study found that iNOS deficiency suppressed cGAS-STING pathway activation in TAC heart, indicating that iNOS may control the activation of the cGAS-STING pathway. Indeed, overexpressing iNOS using a plasmid was sufficient to trigger cGAS-STING activation in cardiomyocytes. Moreover, mtDNA depletion blunted the effect of iNOS on the cGAS-STING pathway. Together, these results revealed that iNOS activated the cGAS-STING pathway via mtDNA. The finding that iNOS is an upstream controlling mechanism may have broad implications in understanding how the cGAS-STING pathway is activated in diseases.

We also confirmed that disrupting the cGAS-STING pathway mitigated sterile inflammation and cardiac remodeling leading to improved cardiac function in pressure overload-stressed heart. The cytosolic mtDNA content significantly increased after 1 week of TAC, whereas a previous study reported that the increase in inflammation occurred after the second day of TAC in mice 33. Thus, inflammation may begin earlier than mtDNA release, suggesting that the cGAS pathway is not the only mechanism underlying the initiation of inflammation.

The results of the present study also provide novel insights into how cGAS-STING activation leads to tissue injury. Similar to the inflammatory responses induced by microorganisms, mtDNA induced sterile inflammation also recruits macrophages to the sites of occurrence in-turn causing tissue damage 40. Accumulation of macrophages may result from the infiltration of circulating macrophages as well as resident macrophages in stressed hearts 28,41. Both cardiac resident macrophages and monocyte-derived macrophages from the circulation contribute to the repair of injured tissues 42. However, infiltrated macrophages within the myocardium may be primed to generate inflammatory responses 43. Macrophage infiltration and activation in TAC heart have been reported to aggravate cardiac dysfunction 44. In the present study, we found that genetic disruption of cGAS or STING blunted M1 polarization and enhanced the proportion of M2 macrophages in TAC heart. These findings showed that the cGAS-STING pathway modulated M1/M2 macrophage polarization in pressure-stressed heart. This is in line with a recent report in which cGAS was found to promote M1 macrophage polarization and aggravate inflammatory responses induced by ischemia 24. Thus, targeting the iNOS-mtDNA-cGAS-STING pathway may shift the pro-inflammatory/anti-inflammatory equilibrium to mitigate cardiac injury induced by mtDNA in pressure overload-stressed heart.

There are limitations that need to be pointed out in this study. Firstly, although we detected no nuclear DNA in cytosol, we could not exclude the effects of DNA in extracellular matrix on cGAS-STING pathway activation. Secondly, we found that iNOS deficiency reduced the cytosolic mtDNA and thus inhibited cGAS-STING pathway, suggesting that iNOS works as the upstream of cGAS-STING pathway. However, how iNOS expression is induced in TAC hearts have not been elucidated. Thirdly, besides mediating inflammation, cGAS also participates many other cell process, such as apoptosis and autophagy 45,46, which further underlies the critical role of cGAS-STING pathway in cardiovascular diseases. However, whether the therapeutic effects of disrupting the cGAS-STING pathway in TAC mice is independent of inflammation has not investigated in the present study.

## Conclusions

**Figure 9:**
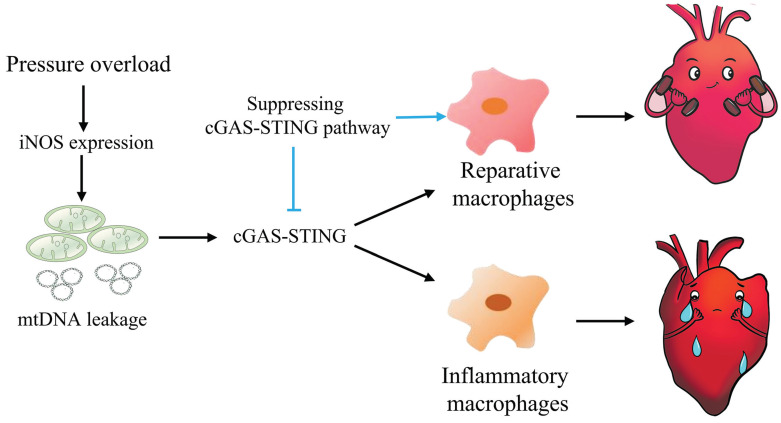
** Graphical mechanistic model.** The model illustrates that pressure overload induces iNOS expression and then activates the cGAS-STING cascade leading to sterile inflammation and cardiac dysfunction in mice.

In summary, this study highlights the concept that mtDNA acts as a DAMP, activating the cGAS-STING cascade and leading to sterile inflammation and cardiac dysfunction in pressure-stressed heart. Inhibition of the cGAS pathway protects heart against adverse remodeling and dysfunction via reduction of the inflammatory responses, likely through enhancing M2 macrophages polarization (Figure 9). In addition, iNOS appears to play a controlling role in aberrant mtDNA release and cGAS activation under pressure overload. Since the increase in iNOS expression as well as cytosolic mtDNA accumulation also occur in hypertensive patients, targeting this cascade may represent a novel approach for treating cardiac remodeling and dysfunction in human diseases.

## Supplementary Material

Supplementary figures and table.

## Abbreviations

cGAS: cyclic GMP-AMP synthase
STING: stimulator of interferon genes
IRF3: interferon regulating factor 3
mtDNA: mitochondrial DNA
WT: wild type
NC: negative control
KD: knockdown
TAC: transverse aortic constriction
ISG: IFN-stimulated genes
iNOS: inducible nitric oxide synthase
DAMPs: damage-associated molecular patterns
PAMPs: pathogen-associated molecular patterns
PRRs: pattern recognition receptors
AAV: adeno-associated virus
LV: left ventricular
EF: ejection fraction
FS: fractional shortening
TNFα: tumor necrosis factor alpha
IL1β: interleukin 1 beta
IL6: interleukin 6
SNAP: S-Nitroso-N-acetyl-DL-penicillamin
IFN: interferon
ANP: atrial natriuretic peptide
BNP: brain natriuretic peptide
Col1: collagen 1
Col3: collagen 3
HHD: hypertensive heart disease
