# Supplementary material for: iNOS aggravates pressure overload-induced cardiac dysfunction via activation of the cytosolic-mtDNA-mediated cGAS-STING pathway
Source: Theranostics. 2023 Jul 24;13(12):4229–46. doi: 10.7150/thno.84049 (PMC10405855; doi:10.7150/thno.84049)
Supplement: Supplementary file 1 — Supplementary figures and table. [file thnov13p4229s1.pdf]

iNOS aggravates pressure overload-induced cardiac dysfunction via activation of the cytosolic-mtDNA-mediated cGAS-STING pathway

Running title: cytosolic mtDNA and cGAS-STING pathway in cardiac dysfunction

Yongzheng Guo[1], Yuehua You[1], Fei-Fei Shang[2], Xiaowen Wang[3], Bi Huang[1], Boying Zhao[4], Dingyi Lv[1], Shenglan Yang[1], Ming Xie[4], Lingwen Kong[4], Dingyuan Du[4], Suxin Luo[1*], Xin Tian[5*] and Yong Xia[1,2,6*]

[1]Division of Cardiology, The First Affiliated Hospital of Chongqing Medical University, Chongqing 400016, China;

[2]Institute of Life Science, Chongqing Medical University, Chongqing 400016, China;

[3]Department of Cardiothoracic Surgery, The First Affiliated Hospital of Chongqing Medical University, Chongqing 400016, China;

[4] Department of Cardiothoracic Surgery, Chongqing University Central Hospital, Chongqing 400014, China;

[5]Department of Neurology, The First Affiliated Hospital of Chongqing Medical University, Chongqing Key Laboratory of Neurology, Chongqing 400016, China;

[6]Davis Heart & Lung Research Institute, Division of Cardiovascular Medicine, The Ohio State University College of Medicine, OH 43210, USA.

***Address for Correspondence:** E-mail: yong.xia@osumc.edu (Yong Xia) ; xintian@cqmu.edu.cn (Xin Tian); luosuxin@hospital.cqmu.edu.cn

This file includes:

Supplemental Fig.1-10

Supplemental Table 1

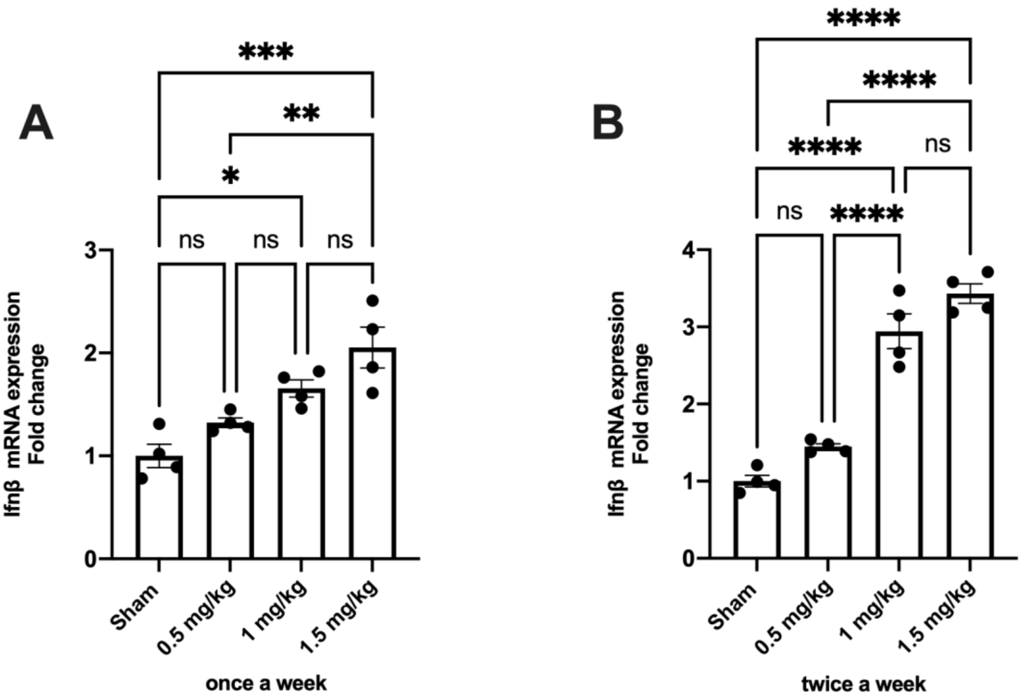

Figure S1. (A-B) mice we subjected to STING agonist at different dosage for once a week or twice a week for 4 weeks. The the IFNβ mRNA expression in heart was measured. The results showed that the dosage of 1 mg/kg STING agonist by intravenous injection twice a week increased the IFNβ expression in heart more effectively, n = 4. Values are means ± SEM. Data were analyzed using a one-way ANOVA with a Tukey's multiple-comparison post-hoc test. *, *P* < 0.05. **, *P* < 0.01. ***, *P* < 0.001. ****, *P* < 0.0001.

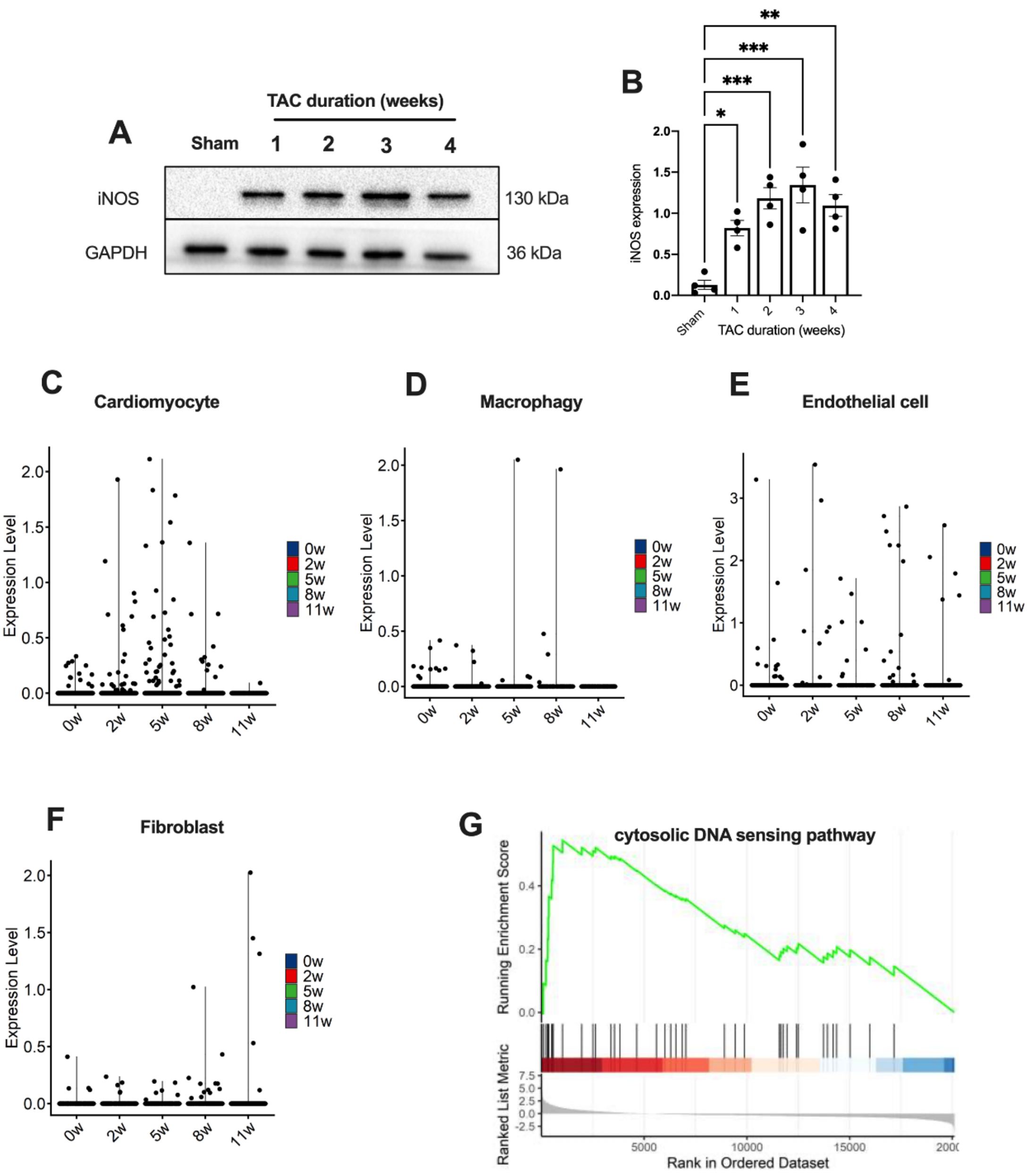

Figure S2. (A-B) Pressure overload increased iNOS expression in the heart, n = 4. **(C-F)** The single-cell sequencing results showed that iNOS expression mainly increased in cardiomyocytes **(C)**, but not in macrophages **(D)**, endothelial cell **(E)** and fibroblast **(F)**. **(G)** Gene set enrichment analysis indicated that the differentially expressed genes were primarily associated with cytosolic DNA pathway in TAC heart. Values are means ± SEM. Data were analyzed using a one-way ANOVA with a Tukey's multiple-comparison post-hoc test. *, $P < 0.05$. **, $P < 0.01$. ***, $P < 0.001$.

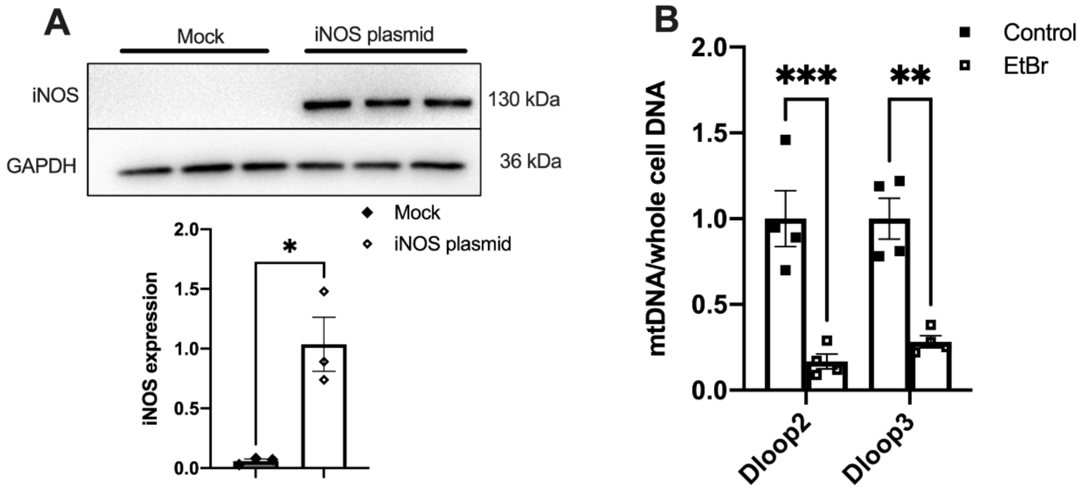

Figure S3. (A) Plasmid encoding iNOS was transfected into isolated cardiomyocytes to overexpress iNOS, and the western blots showed an increased iNOS expression in cardiomyocytes, n = 3; **(B)** EtBr was used to deplete mtDNA in H9C2 cells, n = 4. Values are means ± SEM. Data were analyzed using a one-way ANOVA with a Tukey's multiple-comparison post-hoc test. *, $P < 0.05$. **, $P < 0.01$. ***, $P < 0.001$.

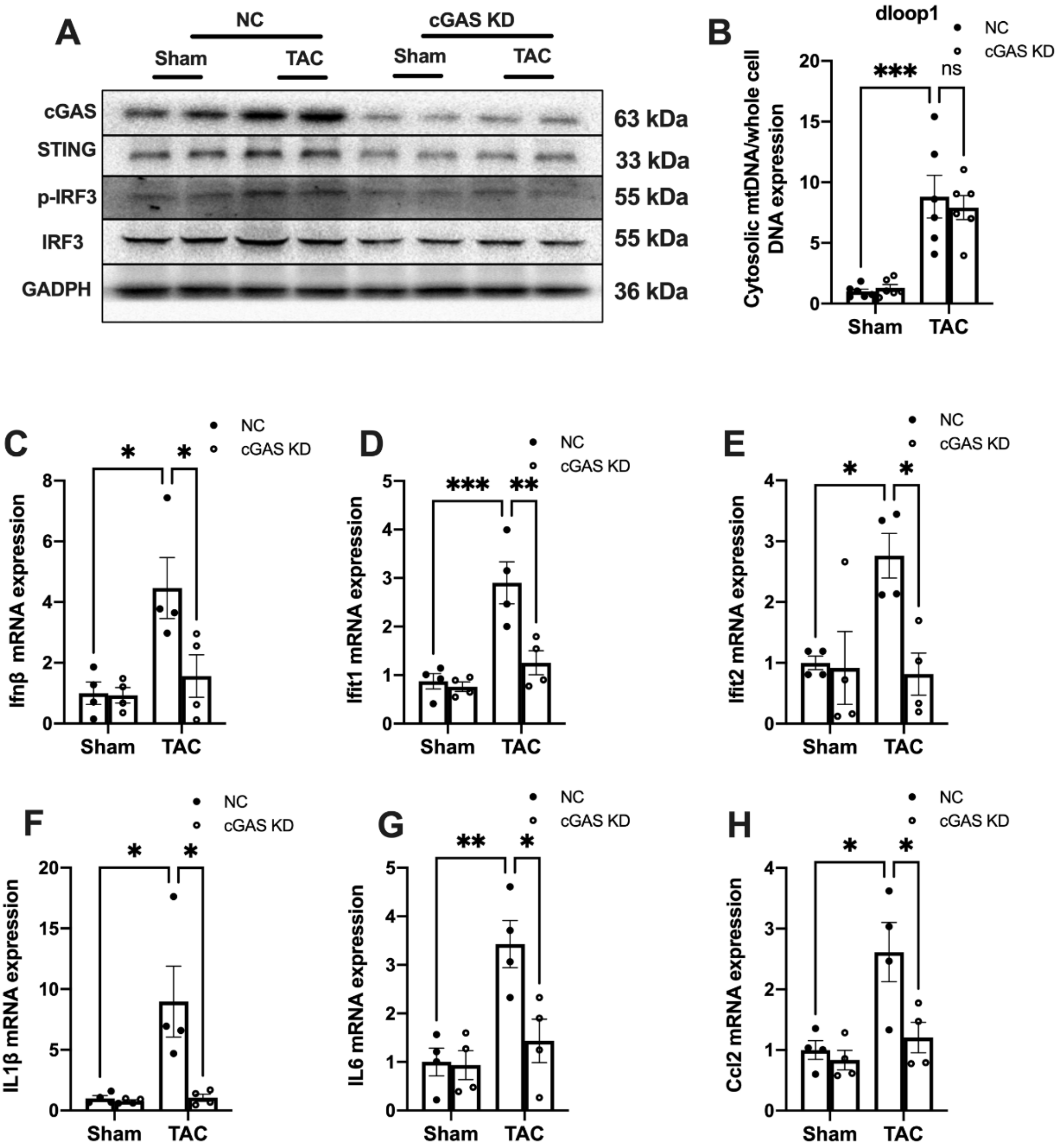

Figure S4. (A) Knockdown of cGAS suppresses the phosphorylation of IRF3 in TAC hearts, n = 6. **(B)** Cytosolic mtDNA content in the heart of TAC mice were measured using quantitative-PCR, n = 6. **(D-E)** Quantitative-PCR was performed to measure the transcript levels of IFN-β and ISGs activated by cGAS-STING, n = 4. **(F-H)** cGAS knockdown suppresses the expression of inflammatory markers in TAC heart, n = 4. Data are presented as the mean ± SEM. Data were analyzed using a two-way ANOVA with a Tukey's multiple-comparison post-hoc test. *, $P < 0.05$. ***, $P < 0.001$. ****, $P < 0.0001$.

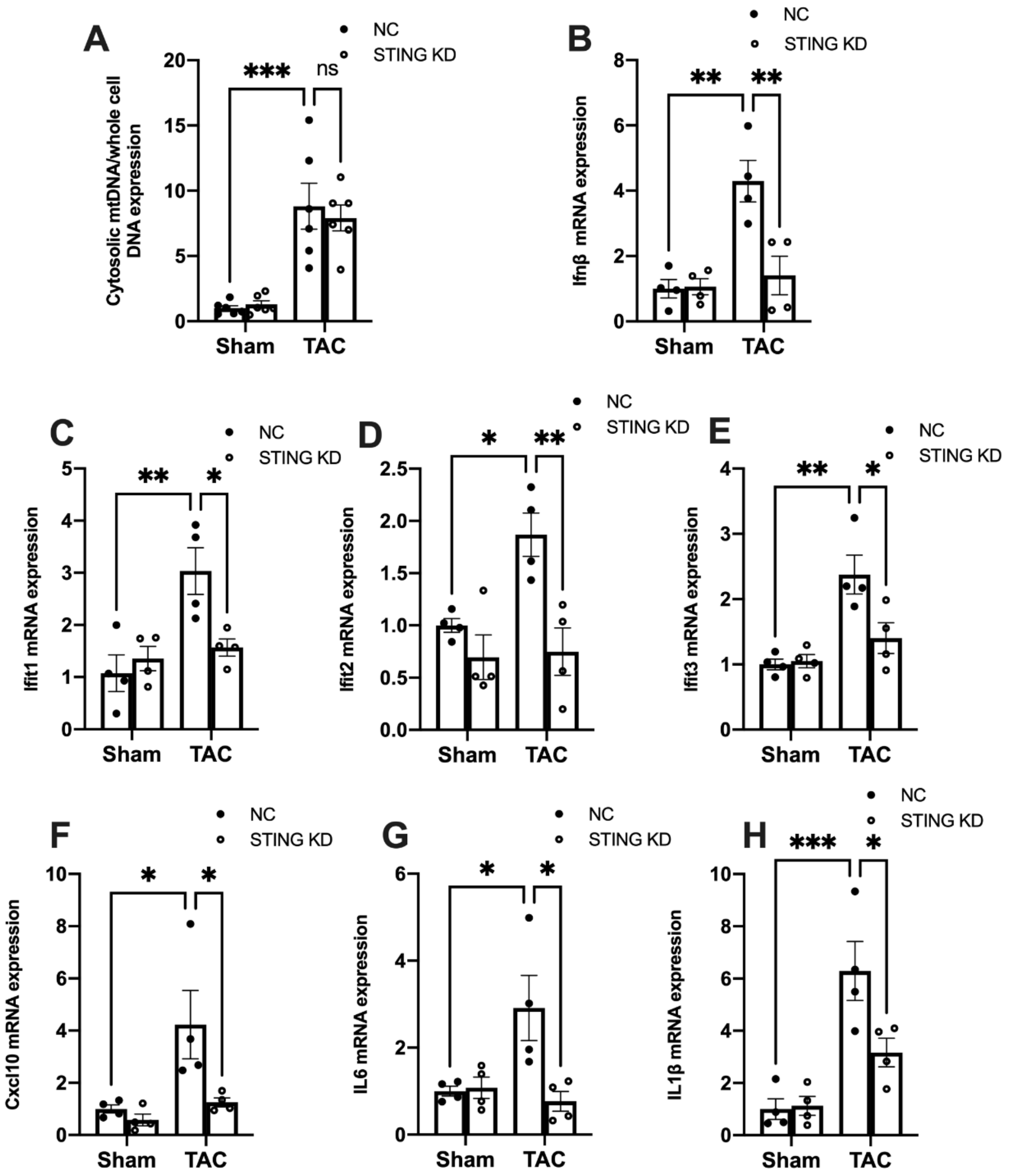

Figure S5. (A) Cytosolic mtDNA content in the heart of TAC mice were measured using quantitative-PCR, n = 6. **(B)** Quantitative-PCR was used to measure the transcript levels of IFN-β activated by cGAS-STING, n = 4. **(D-E)** STING knockdown suppressed the transcript levels of ISGs activated by cGAS-STING in TAC heart, n = 4. **(F-H)** STING knockdown suppressed the expression of inflammatory markers in TAC heart, n=4. Data are presented as the mean ± SEM. Data were analyzed using a two-way ANOVA with a Tukey's multiple-comparison post-hoc test. *, $P < 0.05$. **, $P < 0.01$. ***, $P < 0.001$.

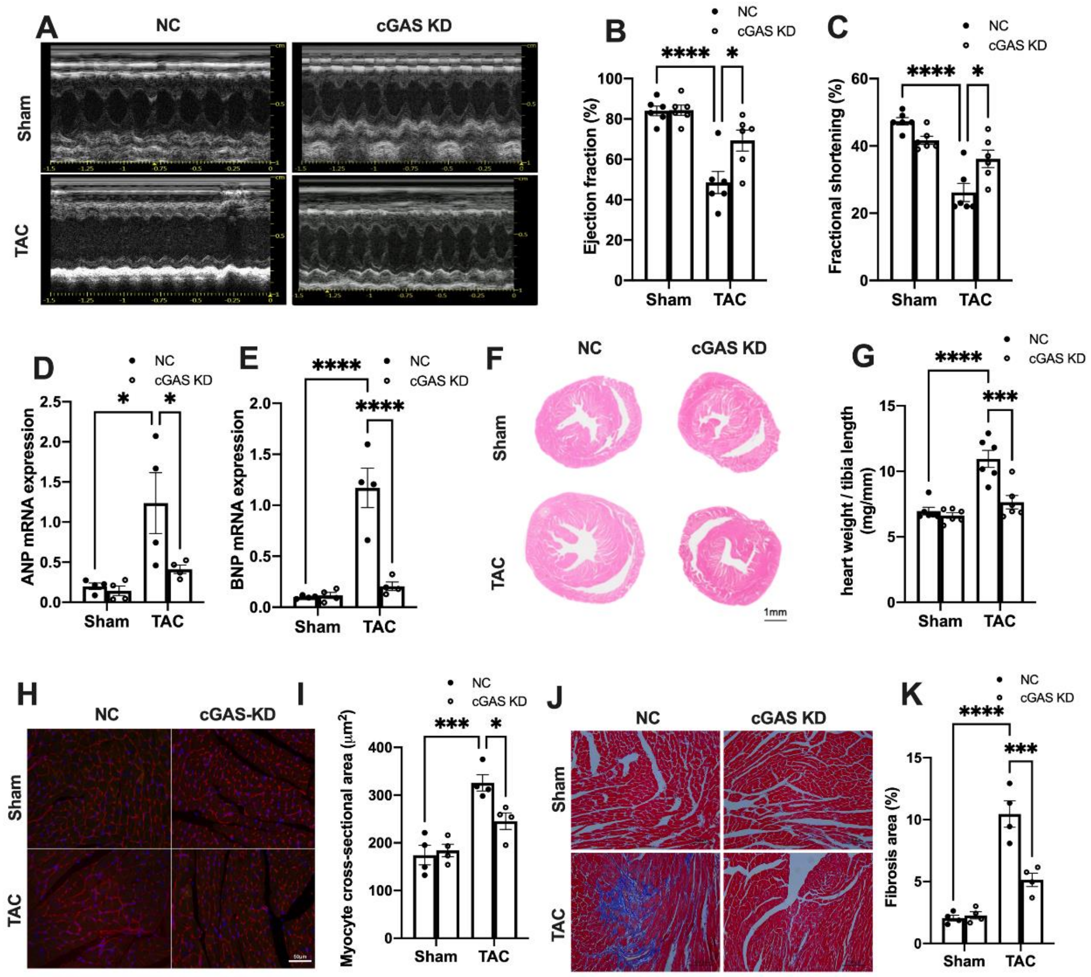

Figure S6. (A) cGAS knockdown improved cardiac function. Representative images of echocardiograms are presented. **(B-C)** LV ejection fraction and fractional shortening are shown in, n = 6. **(D-E)** cGAS knockdown reduced the mRNA expression levels of ANP and BNP in the heart from TAC mice, n = 4. **(F)** Representative hematoxylin-eosin staining of myocardial tissue. Scale bar: 1 mm. **(G)** HW/TL ratio as a measure of cardiac hypertrophy, n = 6. **(H)** Representative WGA staining to assess hypertrophy of cardiac myocytes. Scale bar: 100 μm. **(I)** Quantitative analysis of WGA staining, n = 6. **(J)** Representative images of Masson staining of the heart sections. Scale bar: 100 μm. the results of statistical analysis are shown in **(K)**, n = 4. Data are presented as the mean ± SEM. Data were analyzed using a two-way ANOVA with a Tukey's multiple-comparison post-hoc test. *, $P < 0.05$. **, $P < 0.01$. ***, $P < 0.001$. ****, $P < 0.0001$.

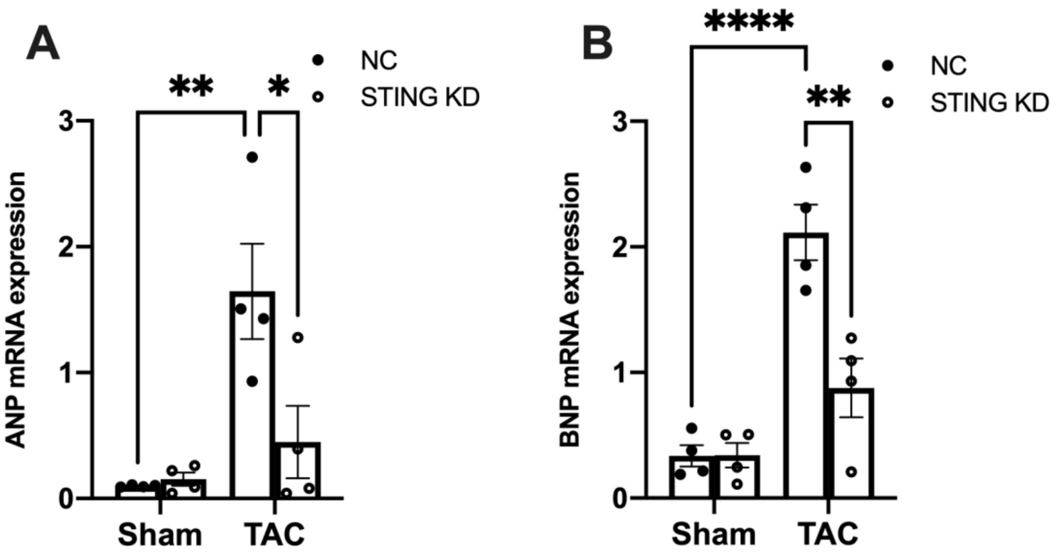

Figure S7. (A-B) cGAS knockdown reduced the mRNA expression of ANP and BNP in the heart from the TAC mice, n = 4. Data are presented as the mean ± SEM. Data were analyzed using a two-way ANOVA with a Tukey's multiple-comparison post-hoc test. *, $P < 0.05$. **, $P < 0.01$. ***, $P < 0.001$. ****, $P < 0.0001$.

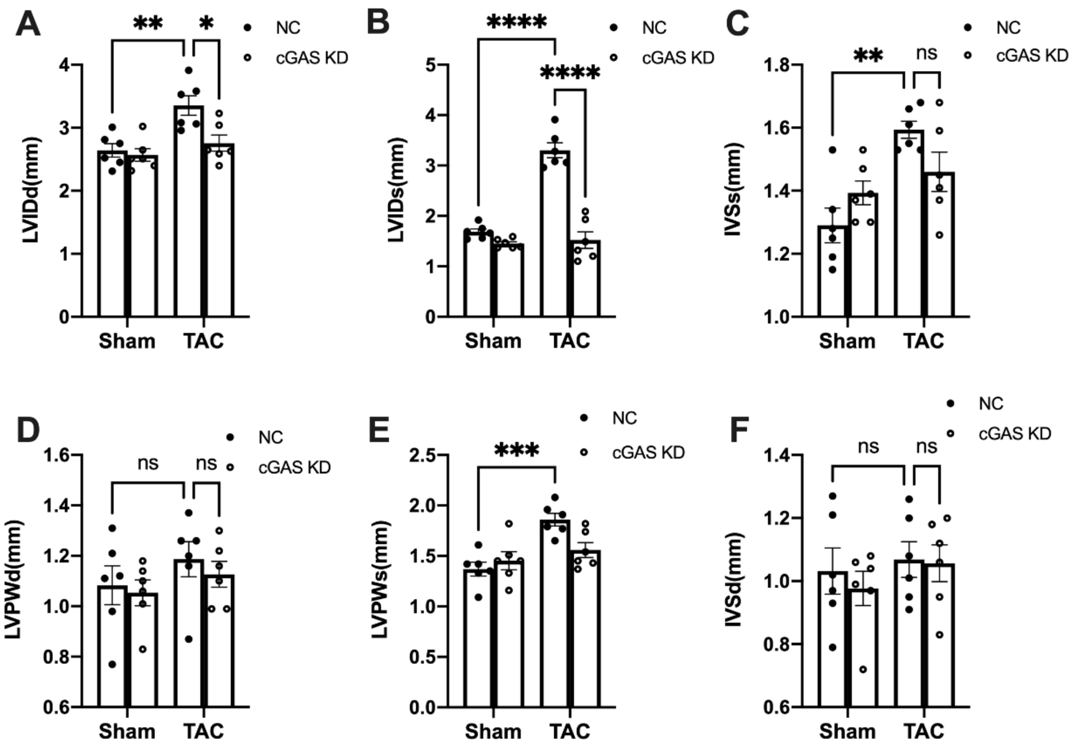

Figure S8. cGAS knockdown protected cardiac function against pressure overload. LVIDd **(A)**, LVIDs **(B)**, IVSs **(C)**, LVPWd **(D),** LVPWs **(E)** and IVSd **(F)** were measured with echo, n = 6. Data are presented as the mean ± SEM. Data were analyzed using a two-way ANOVA with a Tukey's multiple-comparison post-hoc test. LVIDd, end-diastolic left ventricular internal diameter; LVIDs, end-systolic left ventricular internal diameter; IVSd, end-diastolic interventricular septal thickness; IVSs, end-systolic interventricular septal thickness; LVPWd, end-diastolic left ventricular posterior wall thickness; LVPWs, end-systolic left ventricular posterior wall thickness. *, $P < 0.05$. **, $P < 0.01$. ***, $P < 0.001$. ****, $P < 0.0001$. ns indicates no significant.

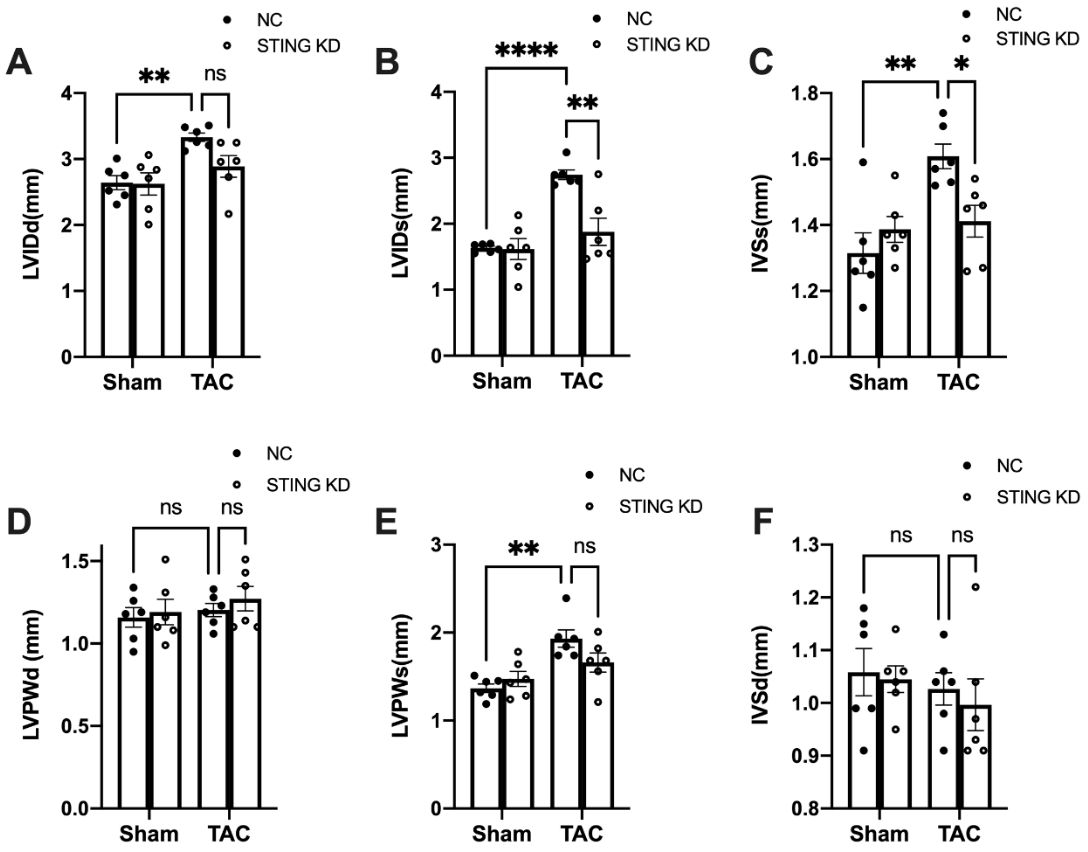

Figure S9. cGAS knockdown protects cardiac function against pressure overload. LVIDd **(A)**, LVIDs **(B)**, IVSs **(C)**, LVPWd **(D),** LVPWs **(E)** and IVSd **(F)** were measured with echo, n = 6. Data are presented as the mean ± SEM. Data were analyzed using a two-way ANOVA with a Tukey's multiple-comparison post-hoc test. LVIDd, end-diastolic left ventricular internal diameter; LVIDs, end-systolic left ventricular internal diameter; IVSd, end-diastolic interventricular septal thickness; IVSs, end-systolic interventricular septal thickness; LVPWd, end-diastolic left ventricular posterior wall thickness; LVPWs, end-systolic left ventricular posterior wall thickness. *, $P < 0.05$. **, $P < 0.01$. ****, $P < 0.0001$. ns indicates no significant.

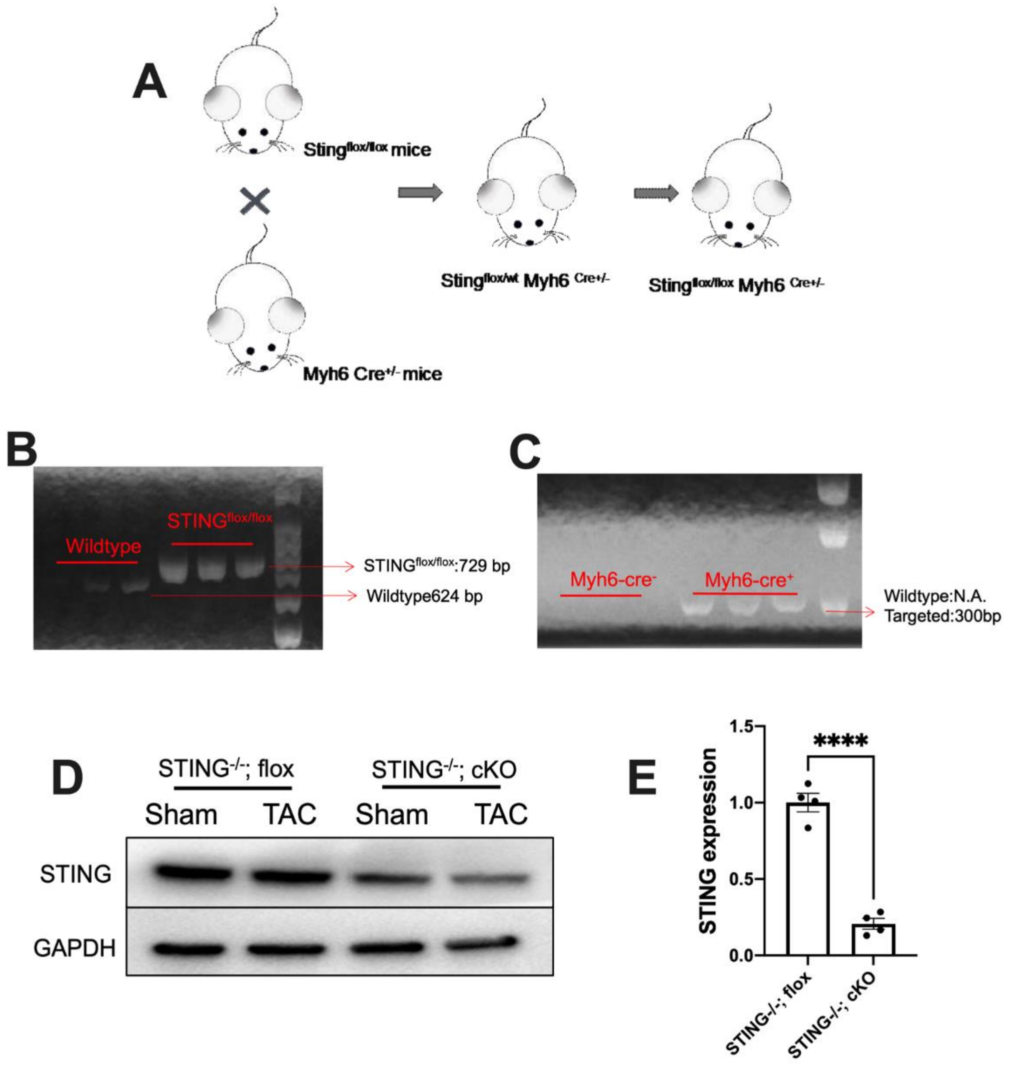

Figure S10. The generation of cardiomyocyte-specific STING deficient mice. **(A)** Schematic view of generation of cardiomyocyte-specific STING depletion mice (STING cKO mice). **(B-C)** Genotyping of STING cKO mice using PCR analysis. **(D-E)** The efficiency of STING depletion was measured with Western Blot, n = 4. Data are presented as the mean ± SEM. Data were analyzed using student *t* test. ****, $P < 0.0001$.

Supplemental Table S1: The used Real-time PCR primers in this study.

Primers	Forward	Reverse
Ccl2	CAGCCAGATGCAGTTAACGC	GCCTACTCATTGGGATCATCTTG
IFNβ1	CTGCGTTCCTGCTGTGCTTCTCCA	GAAGTCCGCCCTGTAGGTGAGGTTG A
IL6	TCCATCCAGTTGCCTTCTTG	GGTCTGTTGGGAGTGGTATC
Cxcl10	TTTCTGCCTCATCCTGCTGGGTCTGA	TGTGCGTGGCTTCACTCCAGTTAAG G
Irf7	GAGCGAAGAGGCTGGAAGACCAACT T	GCAGAACCTGTGTGGGCAGAGCATT
Ifit1	AGGCTGGAGTGTGCTGAGATGGACT G	TGTGCTGCTGAGGGCTTCTTCAATG T
Ifit2	CAGAGGAAGAGGTTGCCTGGAGAGT G	CTTGGTCAGGATGCTGTTGCTGGAT G
Ifit3	CCTGGCACCATGAACCTGAGGACAA C	CCATAAGCAGCACTCCACAGCACAT C
Osal2	TCAGCAGCAGGAAGACCCTAGCAGA T	GCACGGACTCAAGCAGCCAGACTA
GAPDH	ATGGTGAAGGTCGGTGTGAACGGAT T	GTCTCGCTCCTGGAAGATGGTGATG G
ANP	GCTTCCAGGCCATATTGGAG	GGGGGCATGACCTCATCTT
BNP	GAGGTCACTCCTATCCTCTGG	GAGGTCACTCCTATCCTCTGG
Col1	GCTCCTCTTAGGGGCCACT	ATTGGGGACCCTTAGGCCAT
Col3	CTGTAACATGGAAACTGGGGAAA	CCATAGCTGAACTGAAAACCACC
Dloop1	AATCTACCATCCTCCGTGAAACC	TCAGTTTAGCTACCCCCAAGTTTAA
Dloop2	CCCTTCCCCATTTGGTCT	TGGTTTCACGGAGGATGG
Dloop3	TCCTCCGTGAAACCAACAA	AGCGAGAAGAGGGGCATT
